# Duplex-Phase Fe-Mn-Al-C Low-Density Steels: A Review on Their Alloy Design, Processing, Mechanical and Application Performances

**DOI:** 10.3390/ma19050953

**Published:** 2026-03-01

**Authors:** Peng Chen, Yan Lin, Liu-Jiang Yue, Rong Chen, Yi Wang, Ting-Jun Zhang, Xiao-Wu Li

**Affiliations:** Department of Materials Physics and Chemistry, School of Material Science and Engineering and Key Laboratory for Anisotropy and Texture of Materials, Ministry of Education, Northeastern University, Shenyang 110819, China; chenpeng@mail.neu.edu.cn (P.C.); linyan@mails.neu.edu.cn (Y.L.); yuelj@mails.neu.edu.cn (L.-J.Y.); rchen21b@imr.ac.cn (R.C.); wangyinn1999@163.com (Y.W.); 2400688@stu.neu.edu.cn (T.-J.Z.)

**Keywords:** duplex-phase steel, low-density steel, alloy design, processing, performance

## Abstract

Duplex-phase low-density steels are attracting interest for lightweight structural applications, as reducing vehicle mass is an effective route to lower fuel consumption and emissions. This review summarizes recent progress in alloy design, processing, microstructure control, and performance of duplex-phase low-density steels. The roles of major alloying elements are discussed in terms of phase stability and precipitation tendency, followed by an overview of typical processing routes from melting to hot and cold rolling and subsequent heat treatments used to tailor phase fractions and defect structures. Strengthening mechanisms are reviewed with emphasis on precipitation control, including the beneficial contribution of fine intragranular κ′ precipitates and the ductility penalty associated with coarse intergranular κ* films, as well as the use of B2-based particles for high specific strength. Deformation behavior is then discussed in terms of transformation-/twinning-induced plasticity (TRIP/TWIP), planar versus wavy slip, and strain partitioning between ferrite and austenite. Finally, key challenges are outlined, including quantitative interface-based mechanism description, gaps in service property data, stable industrial production and compositional uniformity, and the development of forming and welding windows for engineering implementation.

## 1. Introduction

With increasing pressure to reduce energy use and carbon emissions, lightweighting has become a key strategy in transportation [1]. It has been reported that a 10% reduction in vehicle curb weight can reduce fuel consumption by about 6–8%, supporting higher energy efficiency and lower emissions [2,3]. Therefore, there is strong interest in developing structural materials that combine low density with excellent mechanical properties as alternatives to conventional steels [4]. Against this backdrop, Fe–Mn–Al–C low-density steels have attracted increasing attention as a new class of structural materials [5,6,7,8]. By adding a relatively high amount of aluminum (Al), the density of these steels can be effectively reduced, typically achieving about a 15–25% weight reduction compared with conventional steels [4,5]. Meanwhile, Al addition not only lowers density but, together with manganese (Mn) and carbon (C), can further improve mechanical properties [7]. Mn helps stabilize the austenite phase, while C contributes to strengthening and can also support improvements in strength, toughness and ductility. With proper control of these alloying elements, Fe–Mn–Al–C low-density steels can maintain their lightweight advantage while offering a favorable combination of high strength, good ductility, and high energy absorption, making them promising for transportation and other applications that require low-density yet high-performance materials [9].

Among the various microstructures in Fe–Mn–Al–C low-density steels, ferrite–austenite duplex-phase microstructures are of particular interest due to their attractive mechanical response [6,10]. For the duplex-phase steels, ferrite and austenite exhibit distinct mechanical roles: generally, harder ferrite can significantly increase the yield strength, whereas relatively softer austenite provides good ductility and strain-hardening capacity [11,12]. A well-balanced duplex-phase microstructure can enhance strength while retaining adequate ductility, alleviating the typical trade-off between strength and ductility seen in many conventional steels [10,12,13]. In addition, the cooperative deformation of ferrite and austenite can further improve strain hardening capacity and increase plasticity and toughness during loading. Such stress/strain partitioning between the two phases helps redistribute local stresses and reduce the risk of brittle fracture, enabling a more desirable balance of strength and toughness as well as good formability and reliability for modern engineering structures [14].

At present, the investigation on Fe–Mn–Al–C dual-phase low-density steels has progressed from early alloy composition screening toward more in-depth control of microstructure and properties [15]. Many studies combine thermodynamic calculations with experiments to systematically examine the effects of alloying elements on phase equilibrium, transformation kinetics, and microstructural stability [6,16]. Through tailored thermomechanical processing routes, researchers have been able to more precisely control grain size, phase morphology, and the size and distribution of precipitates, thereby optimizing mechanical performance [17,18,19,20]. However, due to the large design space in composition and microstructure and the involvement of multiple strengthening mechanisms, significant challenges still remain. For instance, key challenges exist in achieving reliable and predictable property control, because strong multiphase coupling makes the composition–processing–microstructure–property relationships highly complex, and precipitation strengthening must be tightly managed to avoid embrittlement. In addition, high Al content complicates large-scale manufacturing, and service properties (e.g., fatigue, corrosion, hydrogen embrittlement, weldability) are still less understood than quasi-static mechanical behavior [15,21].

Based on the above actualities, this review focuses on duplex-phase Fe–Mn–Al–C low-density steels. First, the effects of Al, Mn, and C on phase equilibrium, austenite stability, and dual-phase formation are introduced, together with recent progress in phase-field prediction, alloy design, and microstructure control using CALPHAD-based approaches. Next, the influences of thermomechanical processing and heat treatments (hot rolling, cold rolling, annealing, and aging) on phase fraction, phase morphology, grain size, and precipitation behavior (e.g., κ-carbides and B2 ordered phases) are summarized, with emphasis on typical routes to achieve stable dual-phase structures and controllable precipitation strengthening. Then, key mechanical properties, including strength, ductility, strain hardening, impact toughness, and energy absorption, are reviewed, and the links between microstructural features and properties are discussed in terms of cooperative deformation, interphase boundary effects, and precipitation strengthening mechanisms. In addition, considering engineering application needs, this review also summarizes the current status of service-related properties such as corrosion and stress corrosion cracking, and hydrogen embrittlement susceptibility. Finally, the main open issues are identified, and future research directions and key points for engineering implementation are proposed from the perspectives of alloy design, processing windows, property stability, and manufacturability.

## 2. Alloy Design

The phase constitution of Fe–Mn–Al–C steels is primarily governed by chemical composition and temperature. Achieving a dual-phase (α/γ) microstructure requires the alloy composition and heat treatment window to intersect the ferrite–austenite two-phase region. The location and width of this region, as well as the stability of each phase, are mainly determined by the interactions among the key elements Mn, Al, and C. In addition, microalloying additions are often employed to further refine and stabilize the microstructure to improve overall properties.

### 2.1. Mn

Mn is a key element for in regulating austenite stability, martensitic transformation temperature and the precipitation behavior of κ-carbides, thereby governing the fracture mechanisms and mechanical properties. Song et al. [22] reported that in the Fe-0.8C-*x*Mn-7Al hot-rolled steel, increasing Mn content from 12 to 20 wt% significantly raised the austenite volume fraction from 64.9% to 95.0%. In the low-Mn (12 wt%) alloy, lamellar κ-carbides along austenite grain boundaries promote interfacial separation and early void during deformation, resulting in a poor ductility (2.6% elongation). Conversely, at 15–20 wt% Mn, the reduced grain boundary carbides eliminates these early brittle fracture initiation sites, enabling the austenite matrix to sustain plastic deformation through high-density slip bands and Taylor lattice formation. Notably, the reported strength–ductility synergy (>1 GPa UTS and >22% elongation) was achieved mainly through dislocation substructure refinement, rather than through TRIP or TWIP effects. Mn also influences martensitic substructure evolution in medium-Mn steels. Chen et al. [23] demonstrated that increasing Mn content from 3.06 to 4.64 wt% lowered the martensite start temperature from 365.5 °C to 321.4 °C and refined martensitic lath thickness from 0.32 to 0.29 μm. The dislocation density increased from $1.76\;\times {\;10}^{15}$ to $1.94\;\times \;10^{15}$ m^−2^, which is attributed to the intensified transformation strain. The strengthening of prior austenite by Mn increases the transformation strain and favors thinner laths to accommodate strain relaxation [24]. This evolution significantly enhanced the yield and ultimate tensile strengths through combined solid solution strengthening and martensitic substructure strengthening.

Regarding κ-carbide precipitation, many studies [25,26] suggested that increasing Mn raised the chemical driving force for κ-carbide formation and increased the Mn/Fe ratio within κ-carbides. However, Park et al. [27] observed earlier hardening during aging in lower-Mn specimens (20 wt.%) than in higher Mn content specimens (30 wt%), indicating that κ-carbide precipitation can be delayed by higher Mn. Therefore, the role of Mn cannot be explained merely by a simple substitution of Fe with Mn. Yao et al. [28] proposed that Mn might substitute for Al during κ-carbide formation, accompanied by C vacancies; such vacancies stabilized Mn substitution, and Mn-Al clustering increased the barrier for C atoms to occupy vacancy sites, thereby reducing κ-carbide precipitation. Park et al. [27] further combined experiments with density functional theory (DFT) to show that increasing Mn promoted Mn occupation of Al sites in L1_2_-like supercells, suppressing C ordering through both thermodynamic and elastic strain energy. In particular, C insertion became less favorable energetically, and the lattice expansion upon C incorporation was larger, raising misfit strain energy at the coherent κ/austenite interface. Collectively, by increasing the chemical barrier and lattice distortion penalty, Mn can delay κ-carbide precipitation and associated aging strengthening.

### 2.2. Al

**Figure 1:**
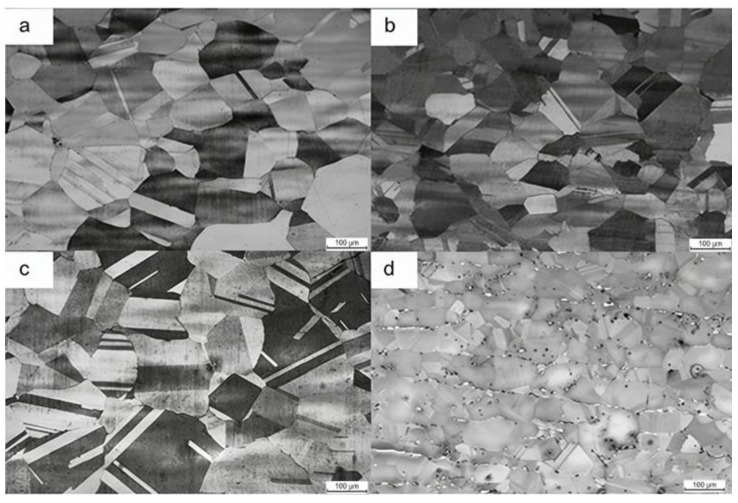
Optical microstructures of Fe–26Mn–xAl–1C steels after solution treatment (1100 °C) and quenching: (**a**) 3Al, (**b**) 6Al, (**c**) 10Al, (**d**) 12Al. (Reprinted with permission from [33], copyright Wiley).

Al is the primary element responsible for weight reduction; per 1 wt.% Al addition leads to 1.3% density reduction [8]. However, extensive Al (often in combination with a high content of C) contributes to the occurrence of brittle intergranular κ-carbides, severely deteriorating ductility [29,30]. In high-Mn lightweight steel, Al elevates the yield strength via solid solution strengthening, but it may reduce the work hardening rate by suppressing deformation twining and shifting the dominant mechanism toward dislocation glide, consistent with its tendency to raise stacking fault energy (SFE) [31,32]. As both a ferrite stabilizer and a κ-carbide-forming element, a small adjustment in Al can strongly influence phase balance and precipitation structures, and consequently the achievable strength–ductility synergy. The microstructures with various Al contents are presented in Figure 1 [33]. Hu et al. [34] identified that in a medium Mn (12 wt%) duplex steel, increasing Al from 6 to 8 wt.% triggered a microstructural transition from single austenite to a multiphase structure (austenite, ferrite, and κ-carbides), improving the yield strength by approximately 200 MPa through precipitation and grain boundary strengthening. On the contrary, in a high-Mn (23 wt%) system, a marginal increase in Al content dramatically shifts the phase equilibrium [35]. Enhancing Al from 9.4 wt% to 12.2 wt% not only elevates the ferrite volume fraction by approximately 3.6 times but also induces the spontaneous precipitation of coarse B2/DO3 phases within the ferrite and brittle β-Mn at the phase boundaries.

### 2.3. C

It is well established that an increase in C content enhances the driving force for the formation of κ-carbides, affecting both intragranular and intergranular precipitation. κ-carbide is a ternary carbide with an E21 structure (Strukturbericht designation), also referred to as a perovskite structure; it can precipitate in austenite as a strengthening phase or form κ-pearlite through a eutectoid transformation with ferrite. Compared with Fe–20Mn–8Al–0.8 (or 1.1)C alloy, Fe–20Mn–8Al–0.4C steel is characterized by coarse κ-carbides at phase boundaries together with a ferritic matrix [27]. Park et al. [27] used ThermoCalc to calculate equilibrium C in ferrite after aging at 550 °C for 1000 min and argued that a lower C content led to a lower ferrite solubility and stronger supersaturation, favoring the formation of coarse κ-carbides.

Liu et al. [36] systematically investigated Fe-11Mn-Al-(0.6–1.2)C steels and linked C content to the precipitation behavior of κ-carbides and the strain hardening mechanisms. In 0.6C alloys, the absence of intragranular κ-carbides resulted in strengthening primarily derived from dislocation interactions within partially recrystallized structures, showing a monotonic decline in the work hardening rate. In contrast, for 1.2C alloys annealed at 1000 °C, a high volume fraction of intragranular κ-carbides triggered a dynamic slip band refining effect, increasing work hardening rate and enabling an excellent strength–ductility synergy. However, high-carbon alloys were strongly sensitive to annealing temperature: at 900–950 °C, eutectoid reaction were accelerated and intergranular κ-carbides formed readily; these coarse κ-carbides were mechanically incompatible with the soft matrix and became preferred microcrack initiation sites.

C also affects recrystallization. Han et al. [37] revealed that higher C notably retarded the austenite recrystallization. At an annealing temperature of 800 °C, the 0.7C steel underwent complete recrystallization, whereas the 1.2C steel retained substantial unrecrystallized regions, exhibiting a bimodal microstructure. The accelerated eutectoid reaction at grain boundaries in high-C steels generated a high fraction of intergranular κ-carbides, which hindered grain boundary migration via the Zener pinning mechanism and thus suppressed nucleation and growth [38]. Although this partially recrystallized structure can yield very high yield strength (1141 MPa), the poor deformation compatibility in unrecrystallized regions combined with the brittleness of coarse intergranular carbides severely reduced the ductility (~9.5% elongation) [37].

### 2.4. Ni

Balaško et al. [39] evaluated the influence of Ni during solidification using thermodynamic calculations and differential scanning calorimetry (DSC). Ni lowered the liquidus, solidus, and austenite formation temperature while preserving the overall transformation sequence and solidification interval. Increasing Ni raised the undercooling for austenite formation, causing smaller dendrites accompanied by finer precipitation morphology in the as-cast specimens. κ-carbides and B2 ordered phase were often discussed in the context of austenite spinodal decomposition. The B2 phase is an ordered body-centered cubic (bcc) intermetallic phase (typically NiAl-type), which often forms in Al-rich ferrite and can markedly influence phase decomposition behavior as well as strengthening mechanisms. Burja et al. [40] investigated Fe-15Mn-10Al-0.8C–(0–8.6)Ni and found that ~5.6 wt.% Ni dramatically facilitated the decomposition of austenite into κ-carbides and B2 ordered phase, whereas 8.6 wt.% Ni retarded the decomposition of austenite and promoted Ni partitioning into ferrite, consistent with the role of Ni as an austenite stabilizer expanding the γ phase domain [41]. Ni was also regarded as an effective SFE-tuning element, increasing SFE by approximately 1.5 mJ/m^2^ per at% [42,43].

Utilizing fine B2 precipitates enabled by Ni addition is a promising route to high-specific-strength steel (HSSS) [44], although strengthening benefits tight control of B2 precipitation [45,46]. Rahnama et al. [47] combined phase-field simulation with experimental characterization for Fe-15Mn-10Al-0.8C duplex lightweight steels, showing that Ni promoted B2 formation but mechanical performance was highly annealing-temperature-dependent (Table 1). At 500–700 °C, Ni-induced ordering produced coarse B2 stringer bands and grain boundary precipitates, which were brittle and prone to cleavage, sharply reducing the ductility [48]. At 900–1050 °C, the competition between interfacial energy and elastic strain energy drove a morphological transition towards nano-sized disk-like particles dispersed within the ferrite matrix, yielding a ductile matrix reinforced with hard NiAl-type B2 and enabling an attractive balance of strength (UTS ~1350 MPa) and ductility (elongation ~22%).

At the atomic level, Ni preferentially bonds with Al rather than Fe, because Ni-Al bonds are stronger than Fe-Al bonds. Breuer et al. [49,50] reported that replacing Fe with Ni yielded a more negative formation enthalpy for the B2 phase than replacing Fe with Al, supporting the thermodynamic preference for NiAl-type B2. Nano-indentation tests combined with density functional theory (DFT) calculations further indicated that stronger Ni-Al bonding increased B2 intrinsic hardness from approximately 8–9 GPa (Ni-free) to 11–13 GPa (Ni-bearing), while Ni also raised austenite hardness by ~1–2 GPa through solid solution strengthening and precipitation strengthening [51]. This multi-scale strengthening mechanism provides a feasible explanation to the observed macroscopic strength increase in Ni-containing lightweight steels.

### 2.5. Si

The addition of Si notably alters the phase transformation thermodynamics and suppresses the carbide precipitation [52]. As a strong ferrite stabilizer, Si raises the austenite transformation temperatures, i.e., Ac_1_ and Ac_3_, and expands the intercritical ferrite–austenite temperature range [53,54,55]. In Fe-10 Mn-3Al-0.2C steel with high Si addition (3 wt.%), a substantial fraction of high-temperature δ-ferrite can be retained, decreasing austenite fraction while increasing C enrichment in retained austenite; this retarded cementite formation improves the chemical stability of austenite [54]. While Si is generally regarded as a non-carbide-forming element in traditional bainitic or TRIP steels [52], it also accelerates κ-carbide evolution in the duplex-phase steel [55]. Sun et al. [55] attributed this to strong Si-C repulsion that drove Si preferentially into austenite, increasing the chemical activity of C within the matrix and enhancing C partitioning from the matrix to precipitates. As a result, κ-carbide nucleation and coarsening may be thermodynamic and kinetically accelerated, contributing to larger precipitates in Si-bearing steel.

From a mechanical perspective, the property response of Si-containing alloys is primarily governed by the combined changes in SFE and austenite fraction [54]. In microstructures with a relatively lower austenite fraction but a higher stability, 1 wt% Si provides stronger solid solution strengthening in ferrite than in austenite [56]. This increases strain partitioning between phases, promotes dislocation multiplication, and can facilitate the activation of TRIP/TWIP mechanisms, thereby improving strain hardening capacity and uniform deformation [54]. However, at excessive Si levels (3 wt%), the ductility may be deteriorated despite the potential enhancement of TWIP (associated with reduced SFE and increased grain size), because coarse and brittle δ-ferrite is introduced. Conversely, in the microstructure with a high austenite fraction but lower stability, the early-stage TRIP response can dominate deformation and mask the Si-induced differences in solid solution strengthening, rendering the macroscopic properties less sensitive to Si content [57].

**Figure 2:**
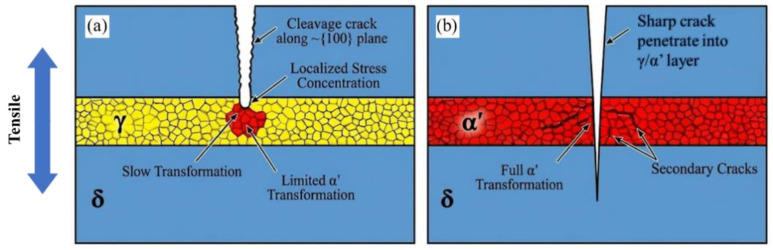
Schematic fracture mechanisms: (**a**) austenite with high stability, corresponding to crack arresting; (**b**) austenite with low stability, corresponding to crack propagation. α′, δ and γ represent martensite, delta-ferrite and austenite, respectively.

Sun et al. [55] further explored the influence of austenite mechanical stability on the fracture mechanisms induced by brittle δ-ferrite in high-Si/Al steels. In Fe-9.7Mn-3.2Al-0.2C-3.4Si steel, cracks are preferentially initiated at δ/γ interfaces due to strain incompatibility associated with the volume change during deformation-induced martensite transformation (DIMT). Cracks then propagate rapidly through brittle δ-ferrite until encountering harder γ/α′ layers; thereafter, retained austenite stability becomes the key factor controlling crack evolution. With high austenite stability, delayed DIMT enables sustained energy dissipation via the TRIP effect at the crack tip, promoting crack tip blunting and arrest and improving total elongation from ~13% to ~33%. With low austenite stability, rapid DIMT produces fresh, brittle martensite prone to secondary cracking and quasi-cleavage fracture, allowing for cracks to propagate through the softer phase layers. Figure 2 schematically compares these two fracture scenarios.

### 2.6. Cu

**Figure 3:**
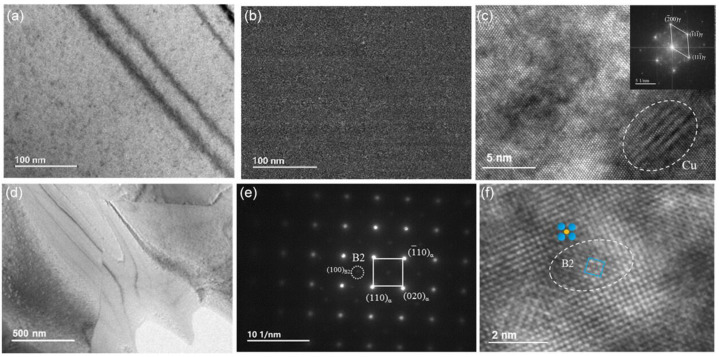
Transmission electron microscope (TEM) images of microstructure in a Fe–9Mn–5.5Al–0.3C–3Ni–1.5Cu steel: (**a**) bright-field image of austenite; (**b**) the corresponding dark-field image of austenite showing the dispersed precipitates; (**c**) high-resolution image of austenite and precipitates showing the Cu precipitate; (**d**) bright-field image of ferrite; (**e**) the corresponding selected area electron diffraction (SAED) showing B2 superlattice; (**f**) high-resolution image of ferrite and precipitates. (Reprinted with permission from [42], copyright Wiley, 2024).

Despite its strong potential for property optimization, systematic studies on Cu in Fe–Mn–Al–C dual-phase lightweight steels still remain limited [58,59,60,61]. Early work on Fe–Cu alloys established that Cu contributed to strengthening through both solid solution hardening and precipitation of ε-Cu [59]. More broadly, Cu-rich precipitates are frequently considered a viable route to increasing strength with a limited ductility penalty, provided that precipitate size, distribution, and interfacial integrity are well controlled [62,63,64]. In duplex-phase lightweight steels, Cu can also act as an austenite stabilizer, increasing both the austenite fraction and austenite grain size over a range of annealing temperatures (typically 730–930 °C) [65]. Distinct from Cu-free alloys, where κ-carbides tend to dominate at lower annealing temperatures (e.g., ~730 °C), Cu-bearing steels can promote the formation of Cu-rich B2-ordered precipitates within the austenite matrix (Figure 3). These precipitates exhibit a B2 superlattice and are enriched in Al and Cu. With increasing annealing temperature, they coarsen while their density decreases. In addition, Cu shows a strong tendency to segregate to interfaces, forming Cu-enriched interfacial layers at γ/α boundaries and at B2 precipitate/matrix interfaces.

Cu addition can further strengthen the alloy through a pronounced solute drag effect by impeding dislocation and grain boundary motion, and Cu delays recrystallization in both austenite and ferrite and thereby contributes to strengthening [66,67,68,69]. Although Cu slightly increases the SFE of austenite (to ~75 mJ·m^−2^), the deformation mode may remain dominated by planar slip and evolve toward a fine Taylor lattice rather than extensive deformation twinning or martensitic transformation [65]. This behavior has been linked to a high yield strength of ~974 MPa and an acceptable work hardening capability, associated with dense dislocation substructures in non-recrystallized regions.

Cu can also interact synergistically with Ni. The combined Cu–Ni effect may facilitate B2 precipitation and improve the strength–ductility balance through multiphase precipitation strengthening together with an increased austenite fraction [42]. Thermodynamic modeling by Stechauner et al. [70] indicated that Ni accelerated Cu precipitation kinetics at 650 °C by reducing incubation time and promoting particle growth, consistent with a low nucleation barrier for coherent Cu and NiAl nanoparticles [45]. At high Ni/Cu and Al/Cu ratios, Ni and Al may segregate to precipitate/matrix interfaces, lowering interfacial energy and further promoting Cu-rich precipitation [42,71]. However, such interfacial segregation may also compromise interfacial cohesion and phase boundary stability, which warrants careful evaluation when targeting precipitation-assisted strengthening.

### 2.7. Cr

Cr addition has been demonstrated as an effective strategy to suppress detrimental intergranular κ-carbides and to refine intragranular κ precipitation, thereby improving ductility while maintaining high strength [72,73,74,75]. In Fe-20Mn-12Al-1.5C steel, Kim et al. [75] reported that adding 5 wt.% Cr significantly retarded coarse κ-carbide formation and increased elongation from 6.1% to 40%. This microstructural improvement is closely linked to how Cr alters carbon solution behavior and κ-phase stability [72]. First-principles calculations and atom probe tomography (APT) disclose that Cr preferentially substitute for Al at corner sites in the E2_1_ lattice, which raises κ formation energy and thermodynamically destabilizes the κ structure [76]. Concurrently, Cr increases the lattice misfit between the κ precipitate and the austenite matrix, thereby elevating both the elastic strain energy and interfacial energy and imposing a higher nucleation barrier. Thermodynamic analyses further suggest that Cr reduces the solution enthalpy of carbon in austenite, expanding carbon solubility and increasing the matrix capacity for interstitial atoms. This reduces the driving force for carbon segregation to grain boundaries, effectively suppressing intergranular κ-carbide formation and leading to a pronounced ductility improvement, with elongation from 14.0% to 47.8% in as-cast conditions [72]. Enhanced initial ductility also benefits downstream processing, enabling an ultra-high strength of ~1.5 GPa with a moderate elongation of ~15% after cold rolling and annealing.

Simultaneously, suppressing intergranular κ-carbides may promote the formation of other carbides such as Cr_7_C_3_. Liu et al. [77] proposed a 5 wt.% Cr design in Fe–30Mn–13Al–1.3C steel, achieving ~1250 MPa tensile strength and ~41.4% total elongation at a low density of 6.36 g∙cm^−3^. They further showed that annealing at 1000 °C dissolved detrimental Cr carbides and drove C/Mn/Al partitioning back into the austenite matrix, removing embrittling grain boundary phases and providing a sufficient chemical driving force for subsequent dispersed intragranular κ precipitation during quenching. Importantly, single-element additions can lead to performance polarization. Cr alone can improve ductility but may also raise the nucleation barrier to the extent that precipitate growth is overly suppressed, yielding only ultrafine short-range ordered (SRO) domains with limited precipitation strengthening. Conversely, Ni alone can markedly increase yield strength by lowering nucleation barriers but may promote κ coarsening, localized planar slip, and reduced ductility [78]. A combined strategy of 2 wt% Ni + 3 wt% Cr has been proposed to balance Ni-driven precipitation tendency against Cr-imposed growth resistance, optimizing κ size to ~5.46 nm and improving the overall strength–ductility synergy [78].

### 2.8. V

As a ferrite-stabilizing element, V annealing at a low temperature (800 °C) promotes the intergranular precipitation reaction (γ→α + κ), leading to a noticeable increase in the ferrite fraction along grain boundaries [79,80]. The poor deformation compatibility between the intergranular α + κ precipitates and the austenite matrix transforms these grain boundaries into preferential sites for crack initiation and rapid propagation, further deteriorating the ductility. Liu et al. [81,82], combining with first-principles calculations, demonstrated that VC particles faced a massive nucleation barrier during low-temperature aging, making the initial high-temperature annealing indispensable for providing the requisite driving force for nanoscale nucleation. Therefore, nanoscale VC particles (1–5 nm) precipitate at an optimal annealing temperature of 900 °C and heavily consume interstitial C atoms, which establishes a competitive relationship with κ-carbides and inhibits their formation [79,80]. The massively dispersed nanoscale VC particles provide substantial precipitation strengthening and grain refinement by pinning dislocations and grain boundaries via the Orowan mechanism, thereby inhibiting abnormal grain growth. However, the dramatic increase in strength is inevitably accompanied by a reduction in ductility compared with the V-free counterpart. The dense network of VC particles impedes dislocation glide and severely restricts the formation of Σ3*^n^*(*n =* 1, 2, 3) boundaries, compromising the capacity for coordinated deformation.

### 2.9. Nb

Minor Nb microalloying can substantially modify precipitation behavior and phase transformation in Fe–Mn–Al–C steels. Zhou et al. [83] investigated 0.02 wt% Nb in Fe–8Mn–6Al–0.15C and found that Nb transformed acicular carbides into spherical nanoscale Nb-containing M_5_C_2_-type carbides. Nb also retarded the γ→α transformation by segregating to phase boundaries, increasing the retained austenite fraction from 30.2% to 35.0% after intercritical annealing at 900 °C. The increased austenite fraction dilutes stabilizing C and Mn within austenite and moderately reduces its thermodynamic stability. This tailored stability enables a more effective TRIP response during deformation, resulting in an improved strength–ductility balance (e.g., ~940 MPa strength and ~36% elongation) compared with the Nb-free alloy.

## 3. Processing Methods

The processing route for Fe-Mn-Al-C duplex steels is designed to manipulate microstructural integrity and phase stability. Typically, following vacuum induction melting, ingots undergo homogenization (1150–1200 °C) and hot rolling (900–1200 °C) to eliminate solidification defects and segregation [20,44,46,51,84,85,86,87,88,89,90,91,92,93,94]. Rapid cooling after hot rolling is essential to prevent undesirable carbide precipitation. Cold rolling has been applied in several processing routes, which introduces substantial work hardening and stored energy within lattice, serving as a thermodynamic driving force for subsequent heat treatments.

### 3.1. Hot Rolling

Hot rolling provides the critical transition from the cast state to a microstructure suitable for subsequent processing; consequently, its process window strongly influences the final property envelope. In practice, homogenized ingots are reheated into the austenite region or the intercritical α + γ region (typically 1050–1200 °C), held for thermal equilibration, and then subjected to multi-pass rolling to the target thickness, followed by controlled cooling or direct water quenching [95]. This sequence is often used not only to shape the material but also to establish phase fractions and defect structures that determine how the alloy responds to subsequent treatments.

Microstructural evolution during hot deformation is governed mainly by deformation temperature, strain rate, and accumulated strain. At higher temperatures, enhanced diffusion, dislocation mobility, and grain boundary migration reduce flow stress and promote dynamic recrystallization, typically yielding fine and comparatively uniform recrystallized grains. As temperature decreases, flow stress rises and dynamic recovery gradually dominates, leading to elongated grains and pronounced deformation substructures [96]. Strain rate modulates the competition between hardening and dynamic softening: higher strain rates generally increase flow stress because dislocation multiplication outpaces softening, which may promote dynamic recrystallization nucleation, and can limit grain growth due to shortened deformation time, thereby contributing to grain refinement [97]. Dynamic recrystallization is typically activated only after the strain exceeds a critical value, and steady-state flow may be reached after peak stress as hardening and softening approach a balance [98]. The coupled effects of temperature and strain rate are commonly rationalized using the Zener–Hollomon parameter (Z), which provides a unified description of hot-deformation kinetics and microstructure pathways.

To optimize the hot-rolling window and avoid flow instabilities such as shear banding, strain localization, and cracking, processing maps are widely employed [97]. By combining power dissipation efficiency with instability criteria, processing maps help identify robust parameter domains that often correspond to the favorable evolution of dynamic recrystallization and an improved processing stability, thereby supporting the development of practical and reproducible hot-rolling schedules.

### 3.2. Cold Rolling

After hot rolling, cold rolling is frequently used to further increase strength and improve dimensional accuracy, particularly for sheet products. Cold rolling imposes severe plastic deformation that profoundly restructures the microstructure: grains become strongly elongated along the rolling direction, dislocation density rises sharply (often to ~10^15^–10^16^ m^−2^), and characteristic deformation substructures such as dislocation cells, walls and microbands develop. Depending on crystallographic orientation and local stress state, deformation twins and shear bands may also appear. Because ferrite and austenite differ in crystal structure, active slip systems, and work hardening behavior, deformation incompatibility between phases can develop during cold rolling, producing local strain heterogeneity and stress concentrations near α/γ interfaces [46].

The most direct outcome of cold rolling is pronounced work hardening: yield strength and ultimate tensile strength typically increase with rolling reduction due to the increased resistance to dislocation motion. This strengthening is usually accompanied by reduced ductility and toughness at higher reductions. Therefore, cold-rolled material is commonly used as a pre-conditioned state for subsequent heat treatment, where controlled recovery and recrystallization restore ductility while enabling phase tailoring and precipitation control for comprehensive property optimization.

### 3.3. Heat Treatment

**Figure 4:**
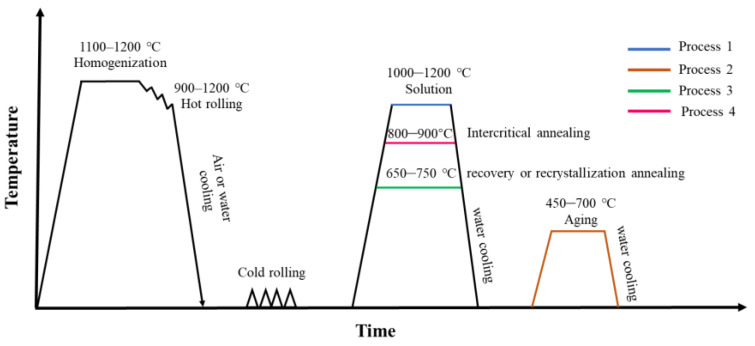
General process for producing duplex lightweight steel. Processes 1–4 represent distinct routes for heat treatment.

Heat treatment is used to reset phase distributions, tune the dual-phase balance, and activate precipitation strengthening; as illustrated in Figure 4, commonly used schedules can be grouped into four representative routes. Solution treatment at 1000–1200 °C is applied to dissolve secondary phases and regulate phase fraction and distribution. A subsequent aging step (typically 450–700 °C) is used to strengthen the matrix via controlled precipitation. Recovery/recrystallization annealing at ~650–750 °C can be selected to partially retain deformation substructure—preserving strength while restoring ductility. Intercritical annealing around ~800–900 °C is widely used to tailor ferrite and austenite fractions and their stability to achieve optimized dual-phase properties.

**Figure 5:**
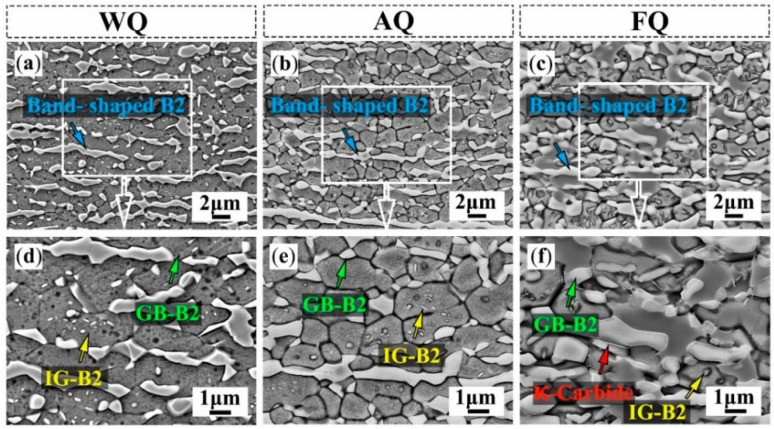
Scanning electron microscope (SEM) images: (**a**,**d**) WQ sample, (**b**,**e**) AQ sample, and (**c**,**f**) FQ sample. (Reprinted with permission from [102], copyright MDPI, 2025).

The precipitate strengthening via aging treatment strongly relies on the heat treatment temperature, cooling pattern, and isothermal time. Aging temperature strongly dictates κ-carbide evolution. Below ~600 °C, sluggish partitioning can leave Mn and C insufficient to stabilize austenite, promoting austenite decomposition and the formation of fine lamellar κ-carbides within the austenite matrix [40,99]. Around ~700 °C, κ-carbides in austenite may be spheroidized and partially dissolved; the associated solute redistribution can promote κ-carbide nucleation along ferrite grain boundaries, which is often detrimental to ductility. However, for duplex lightweight thick plates steel intended for marine structures and cryogenic storage vessels, the traditional sheet manufacturing route, e.g., cold rolling followed by final annealing, is physically and economically impractical. Yang et al. [100] demonstrated that thick hot-rolled duplex lightweight steel plate with a composition of Fe-15Mn-6Al-(0.2, 0.4)C subjected to low-temperature aging (below 600 °C) was a viable heat treatment to further enhance its properties. At this point, κ-carbide begins to precipitate within the austenite, consuming C and Mn from the matrix. The local depletion of C and Mn directly lowers the SFE of the austenite, thereby triggering the Transformation-induced plasticity (TRIP) effect and significantly elevating the work hardening rate. Cooling rate after high-temperature exposure is equally critical; slow cooling generally encourages grain boundary precipitation and coarsening (e.g., coarse B2 and κ phases), and is therefore typically unfavorable for ductility retention [8,101]. For Fe-18Mn-10Al-1C-5Ni steels, furnace quenching (FQ) was reported to promote B2 phase (GB-B2) and κ-carbide along austenite grain boundaries, whereas water quenching (WQ) and air quenching (AQ) effectively suppressed the grain sizes of both the austenite and B2 phases as well, leaving uniformly dispersed nanoscale intragranular B2 particles (IG-B2) and improving elongation from ~2.3% to ~23.3% [102]. The resulting microstructures under different cooling conditions are summarized in Figure 5. Aging time can further shift the balance between strengthening and embrittlement. In Fe–11Mn–10Al–1.2C aged at 550 °C, rapid coarsening of nanoscale intragranular κ-carbides at short times (3–10 min) increases the yield strength, whereas prolonged aging (30–240 min) promotes eutectoid reactions at grain boundaries and may introduce Widmanstätten ferrite. Although these transformations can further raise the yield strength, they often cause a severe loss of ductility [99].

**Figure 6:**
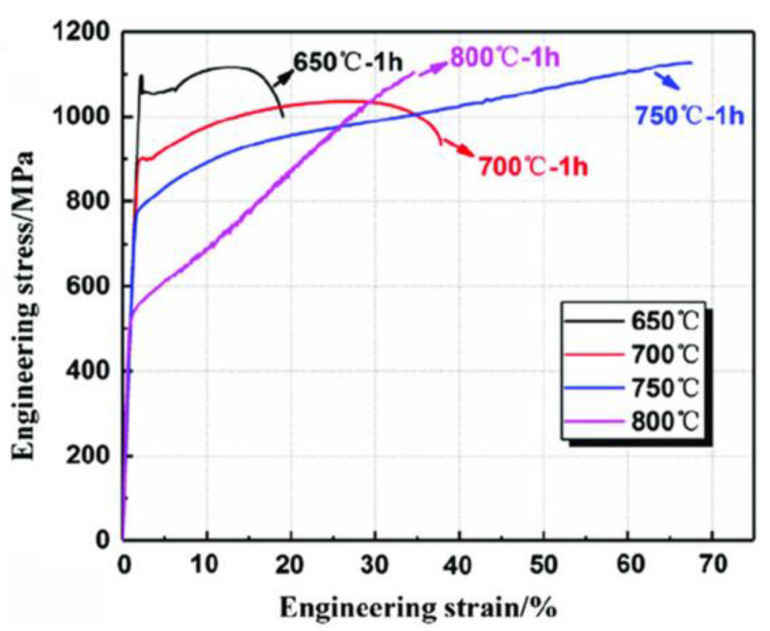
Engineering stress–strain curves of Fe-7Mn-3.2Al-0.4C steel intercritical annealed at different temperatures for 1 h. (Reprinted with permission from [107], copyright Wiley, 2019).

The dependence of mechanical properties on intercritical annealing temperature has been extensively investigated [61,103,104,105,106,107]. As illustrated in Figure 6, lower intercritical temperatures (approximately 800–900 °C) can promote refined multiphase architectures (often reported as δ + α + γ under certain conditions), in which partitioning enhances austenite stability and improves the strength–ductility synergy. Increasing the temperature toward ~1000 °C can eliminate α-ferrite and coarsen austenite grains, reducing mechanical stability and increasing susceptibility to early fracture, typically accompanied by a drop in yield strength. Accordingly, an intercritical window around 800–850 °C is frequently proposed as a practical range to maximize structural refinement while maintaining favorable phase stability.

Beyond conventional schedules, several emerging strategies have been proposed to overcome traditional trade-offs [61]. Sohn et al. [108] proposed that cold rolling followed by annealing at 650–700 °C created a duplex structure of ultrafine ferrite and elongated, non-recrystallized austenite; by exploiting TRIP, this microstructure achieved an UTS ~1.52 GPa together with a total elongation ~49.5%. Liu et al. [42] combined centrifugal casting (cooling rates ~10^2^–10^3^ K·s^−1^) with cold rolling to suppress segregation and refine the as-cast structure. Warm rolling at 500–600 °C has also been introduced to utilize deformation-induced ferritic transformation, refine grain size, and stabilize austenite, thereby suppressing premature strain-induced martensite transformation and improving cold workability [109].

### 3.4. Other Advanced Processing Technologies

In addition to conventional hot rolling, cold rolling, and heat treatment, advanced processing techniques are being explored to further enhance performance or address specific manufacturing challenges. Traditional technologies for fabricating Fe–Mn–Al–C duplex steels, involving in casting, rolling and annealing, is expensive and time-consuming [110]. Laser powder bed fusion (L-PBF), utilizing a high-energy laser beam to induce rapid heating and melting of metal powder, has become an alternative manufacturing option. Li et al. [111] disclosed that the exceptional mechanical performance of L-PBF fabricating Fe–10Mn-1.6Al-0.4C-0.7V steel is fundamentally dictated by an extremely high initial dislocation density (9.7 × 10^14^ m^−2^), which originates from the complex physical metallurgy of the layer-by-layer printing process. The severe thermal gradients, cyclic rapid cooling, and subsequent remelting of previously solidified layers simultaneously trigger localized recrystallization and generate significant internal stresses, which establishes a dense dislocation network within the ultrafine martensitic laths. Although Al is vital for weight reduction, the excessive Al content poses a threat to formability and processability, i.e., bringing challenges to generate parts with complex geometries [110,112]. Xie et al. and Yan et al. [110,113] induced L-PBF to break those limits and primarily investigate the influence of laser scanning speed. The rapid solidification rates inherent to L-PBF suppress solute segregation, stabilizing a fully austenite matrix across all tested scanning speeds from 280 mm/s to 1400 mm/s. Increasing the scanning speed elevates the cooling rate and undercooling, which decreases the critical nucleation radius and systematically refines the equiaxed grain size from 13.1 µm down to 7.9 µm. Concurrently, grain orientation transitions from a distinct <101> texture at 800 mm/s to a strong <001> texture oriented opposite to the heat flow direction at 1400 mm/s. Köhnen et al. [114] explored how the addition of Al govern the solidification behavior and mechanical properties in a Fe-21Mn-(0~5.4)Al-0.3C steel processed via L-PBF. The 0~3Al alloy predominantly undergoes an austenitic solidification pathway. Driven by the steep thermal gradients inherent to L-PBF, it displayed severe epitaxial grain growth characterized by coarse columnar grains and a pronounced <101> crystallographic fiber texture parallel to the build direction. Conversely, increasing the Al content to 4–5.4 wt% acts as a body-centered cubic (bcc) stabilizer, fundamentally shifting the solidification sequence to a ferritic–austenitic pathway. This sequence initiates with primary bcc formation, followed by secondary face-centered cubic (fcc) nucleation in solute-enriched intercellular regions, and concludes with a partial solid-state bcc-to-fcc transformation during rapid cooling. The phase transformation effectively disrupts the epitaxial growth mechanism, drastically altering the microstructure into a dual-phase structure with a nearly random crystallographic orientation.

Direct energy deposition (DED) is another additive manufacturing technique, characterized by directly injecting feedstock powders into a melt pool on the substrate [115,116]. Compared with L-PBF, it permits independent multi-powder feed control and in situ alloying [117]. Nam et al. [118] fabricated duplex lightweight steels via DED unprecedentedly, achieving flexible tuning of Al (8–12 wt%) and Mn (20–25 wt%) contents. Their study demonstrates that the rapid solidification and severe thermal cycling inherent to the DED process decisively dictate the phase transformation pathways. This not only drives a primary matrix transition from austenite (8Al) to ferrite (10Al and 12Al) but also induces morphological variations in the secondary phases driven by disparate diffusion kinetics. In low-Al-addition alloy, C segregation at dislocation networks creates local chemical heterogeneities, which anomalously trigger the twinning-induced plasticity (TWIP) mechanism within an austenitic matrix that otherwise possesses a relatively high theoretical stacking fault energy. However, the DED in situ alloying of high-Al systems also exposes critical limitations. In the 12Al composition, the detrimental coupling of DED-induced residual stresses with brittle, network-like κ-carbides precipitated along grain boundaries, alongside B2 and L2_1_ ordering within the matrix, leads to catastrophic microcrack propagation and premature failure before yielding.

Friction stir processing (FSP) is a solid-state surface modification method in which a rotating tool generates frictional heat and severe local plastic flow, leading to intense microstructural refinement. For duplex lightweight steels, FSP has the potential to generate ultrafine-grained or even nanocrystalline surface layers for improved surface hardness and wear resistance, heal casting-related defects such as pores and microcracks, and locally tailor phase constitution by promoting solid-state transformations [119]. Although current research remains at an early stage, FSP appears promising for localized strengthening and defect remediation.

Electropulsing treatment is another emerging approach, applying high-density transient current pulses that can introduce electron wind forces, enhance atomic diffusion, and accelerate dislocation motion and annihilation. Reported benefits include enabling recrystallization at lower average temperatures to mitigate grain coarsening, refining and dispersing precipitates, and reducing internal defect density to improve ductility and toughness with limited sacrifice in strength [120].

While these approaches require further systematic studies, particularly regarding process windows, scalability, and structure–property reproducibility, they offer additional pathways for non-conventional microstructure control in Fe–Mn–Al–C dual-phase lightweight steels.

## 4. Strengthening Mechanisms

### 4.1. Precipitation Strengthening

#### 4.1.1. κ-carbide Precipitation and Strengthening

κ-carbide is the characteristic strengthening precipitate in Fe–Mn–Al–C low-density steels. It adopts an ordered E2_1_ (perovskite-type) structure in which Al occupies the cube corners, Fe/Mn occupies the face centers, and C resides at the octahedral interstitial site coordinated by six nearest-neighbor Fe/Mn atoms [19,78,121,122]. A perfectly stoichiometric (Fe,Mn)_3_AlC composition is rarely observed in practice; experimentally measured κ-carbides typically deviate from ideal stoichiometry due to off-stoichiometric partitioning and point defects [19,26,28,123]. Because Mn substitutes on the Fe sublattice in the fcc-related framework, κ-carbide forms a compositional continuum from Fe_3_AlC toward Mn_3_AlC, and increasing Mn content generally expands the κ lattice parameter, consistent with the larger atomic radius of Mn relative to Fe [124].

**Figure 7:**
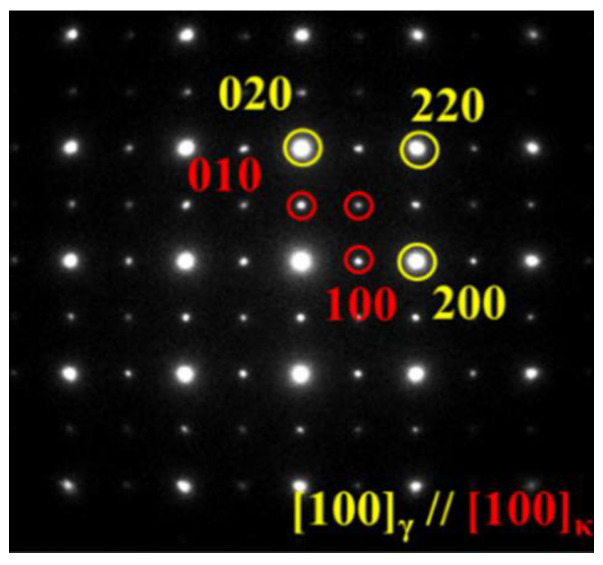
Selected area electron diffraction (SADP) pattern of the κ′-carbides along [100]_γ_ orientation, which presents a coherent with γ. Adopted from Ref. [138].

From the viewpoint of morphology, interfacial character, and mechanical consequences, κ-carbides are commonly classified into intragranular κ′ and intergranular κ* [8]. Intragranular κ′ precipitates form within the austenite matrix and are typically nanoscale and highly coherent because the κ′/γ lattice misfit is small (often reported < 2%) [123,125]. Accordingly, κ′ maintains a cube-on-cube orientation relationship with austenite, e.g., [100]κ//[100]γ and (001)κ//(001)γ (Figure 7) [126,127,128,129]. In contrast, κ*-carbides preferentially decorate austenite grain boundaries and may also appear in ferrite, where they are generally coarser and lose coherency and a well-defined orientation relationship; the κ*/matrix interface becomes sharp and mechanically weak, as evidenced by distinct boundary contrast [130,131]. When κ forms in ferrite, the larger crystallographic mismatch is particularly unfavorable; misfits on the order of ~5.9% have been reported, and direction-dependent misfit values (e.g., ~6.67% along [011]κ//[001]α) help explain the tendency toward elongated/rod-like κ morphologies in ferrite [132,133]. This coherency contrast is central to property optimization: fine coherent κ’ is generally strengthening-favorable, whereas coarse incoherent κ*, especially as continuous grain boundary films or coarse bands, tends to promote strain localization and premature failure [99,134,135,136,137].

**Figure 8:**
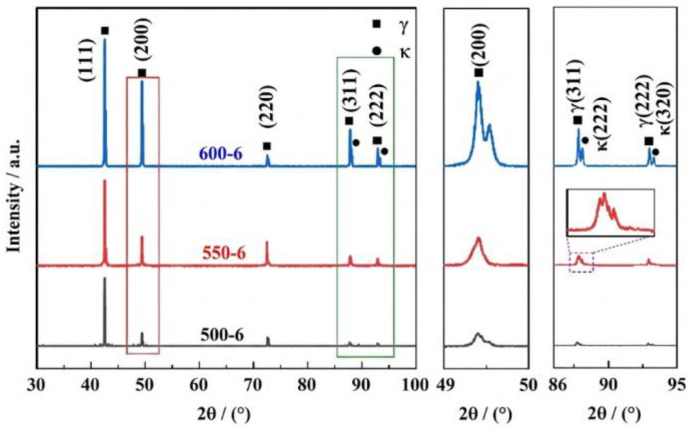
XRD patterns of a Fe-30.5Mn-8Al-1.0C aging for 6 h at different temperatures: the specimen with the precipitation of κ’-carbide, which displays a broadening of the austenite peak. (Reprinted with permission from [143], copyright Elsevier, 2023).

Because κ′ is often extremely fine, conventional laboratory X-ray diffraction (XRD) analysis may not resolve separate κ′ reflections unless the volume fraction becomes sufficiently high. Instead, early-stage κ′ formation is commonly inferred from austenite peak broadening associated with spinodal decomposition and compositional modulation [139,140,141,142]. The basic signature is a two-sided distortion of the γ peak: C-rich, κ′-precursor domains expand the local lattice and extend intensity toward lower 2θ, while C-depleted γ contracts and shifts the main peak toward higher 2θ (Figure 8) [141,143]. With increasing κ′ fraction and misfit, peak broadening can evolve into peak splitting, reflecting the emergence of distinguishable precipitate/matrix lattice parameters. By contrast, the appearance of distinct superlattice reflections at specific angles that do not correspond to coherent fcc positions (e.g., around 2θ ≈ 34.3° in some reports) is typically associated with coarse incoherent precipitation, most notably intergranular κ* [138]. In practice, combining diffraction with TEM and local chemical analysis is often necessary to unambiguously separate “coherent κ′ strengthening” from “incoherent κ* embrittlement”.

The precipitation of κ-carbide in Fe-Mn-Al-C steels is highly sensitive to both composition and thermal history and proceeds through two broadly different microstructural routes: (i) boundary-associated discontinuous/cellular transformations and (ii) intragranular spinodal decomposition followed by ordering. Boundary-driven discontinuous precipitation involves migration of a reaction front from grain boundaries, leaving lamellar colonies of κ* and solute-depleted γ; at relatively low temperatures (around ~600 °C), limited driving force and slow long-range diffusion favor coarse lamellae that provide little strengthening because dislocations can bypass them readily [144,145,146]. In alloys with relatively high Al contents (>5 wt%), a related boundary-associated precipitation transformation has been reported near ~600 °C, where κ* may first appear as discrete boundary particles and subsequently evolve into a more continuous lamellar boundary structure [40,145,146]. With higher temperatures (e.g., ~800 °C) or prolonged holding around ~650 °C, cellular transformation becomes prominent, producing widely distributed lamellar colonies such as α + κ (or α + κ + γ’) [40,147,148]. This is often described as a eutectoid-type reaction γ→α + κ driven by austenite instability; segregation of C and Al toward grain boundaries creates solute-depleted regions that promote cooperative lamellar growth of ferrite and κ at boundaries [145,146,149]. In contrast, the beneficial nanoscale intragranular κ′ population is generally linked to spinodal decomposition, where compositional fluctuations partition austenite into solute-lean (γ′) and solute-rich (γ″) regions. The solute-rich regions first develop L1_2_-type (Fe,Mn)_3_Al ordering and subsequently evolve into κ′ as carbon orders into octahedral interstices, often summarized as [150,151,152,153]

(1) $$\gamma \to \gamma^{\prime }+\gamma^{\prime \prime }$$

(2) $$\gamma^{\prime \prime }\to L1_{2}$$

(3) $$L1_{2}\to \kappa$$

These distinct routes naturally lead to very different morphologies and, therefore, very different mechanical outcomes [40,145,146].

**Figure 9:**
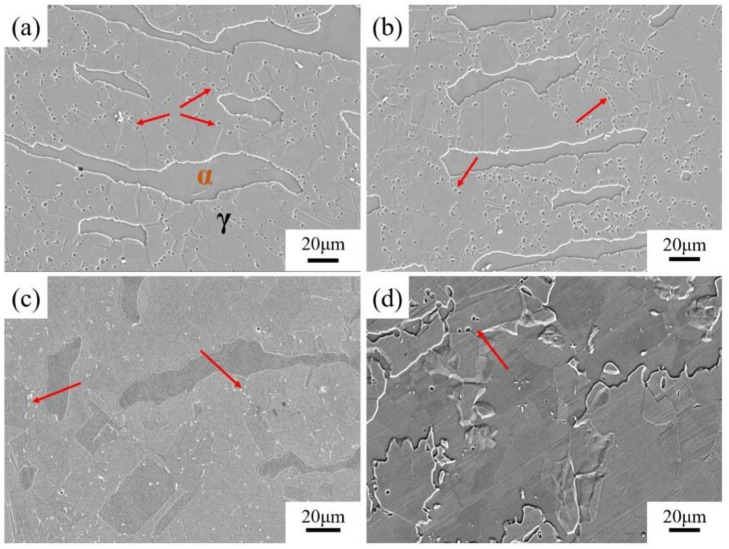
SEM images of the microstructure in solid solution specimens at different temperatures: (**a**) 800 °C, (**b**) 850 °C, (**c**) 900 °C, and (**d**) 950 °C (red arrows indicate discrete particles). (Reprinted with permission from [157], copyright MDPI, 2024).

A useful way to rationalize heat treatment sensitivity is to consider three broad temperature regimes (especially for cold-rolled duplex microstructures) [40,99,154]. Below ~600 °C, intragranular spinodal decomposition competes with boundary cellular reactions; higher austenite stability tends to suppress eutectoid decomposition and favor κ′ formation, whereas reduced stability promotes boundary reactions. Between 600 °C and 750 °C, many alloys exhibit an unfavorable transition where metastable intragranular precipitates can dissolve or coarsen while solute segregation intensifies, increasing the propensity for detrimental intergranular κ* precipitation and associated embrittlement [155]. At higher temperatures approaching 900 °C, the austenite stability increases and κ-carbides tend to dissolve (often described by κ + α→γ), potentially driving the microstructure toward a fully austenitic state in some compositions [99]. The microstructure evolution with temperature has been summarized in Figure 9. In addition to temperature, stored energy plays a key role: greater prior deformation (i.e., higher dislocation density) can accelerate κ precipitation by reducing nucleation barriers and providing rapid diffusion paths, thereby altering both kinetics and precipitate distributions [5,149,156].

The thermodynamics of κ-carbide precipitation are dominated by the solute concentrations of C and Al [25,27,28,40,153]. C generally stabilizes austenite while also providing the chemical driving force required for κ formation, so its influence is intrinsically dual: it can delay austenite breakdown by stabilizing γ yet simultaneously promote κ precipitation once ordering/partitioning becomes favorable [36]. Although Al is a ferrite stabilizer in the matrix, it expands the κ stability domain and can promote eutectoid decomposition, owing to its strong participation in the chemistry of κ and its segregation at boundaries [132,158]. Extensive investigations have established that spinodal decomposition can proceed even during the water quenching following solution treatment, which has been attributed to the substantial driving force associated with C supersaturation in austenite [40,158,159,160,161]. Concerning κ solvus temperature, Kim et al. [131] noted that κ precipitation can persist above values predicted by an empirical relation, *T*_κ_ (K) = 1010 + 22(Al wt%) − 4(Mn wt%), implying that the actual κ stability may be underestimated by simple composition-only estimates in some conditions [153]. In practice, elevated Al and C broaden the κ stability window and can sustain decomposition even under relatively rapid cooling, whereas insufficient Al/C reduces the driving force for eutectoid reactions and can shift the system toward boundary-associated discontinuous precipitation with lamellar κ* and poor ductility [108,137,162]. High Mn contents (often >~12 wt%) can markedly delay ordering kinetics by obstructing carbon ordering into octahedral interstices, which may require longer aging to develop fine κ′ but can be beneficial in suppressing coarse κ* formation [27,28,36,161].

Mechanically, κ-carbides can raise the yield strength through different routes, but their ductility impact depends strongly on morphology and location. Lamellar eutectoid colonies (often termed κ-pearlite, α + κ) can provide strong strengthening by efficiently impeding dislocation motion; compared with conventional θ-pearlite (α + Fe3C), κ-pearlite may exhibit finer interlamellar spacing because sluggish Al diffusion limits lamellar thickening, consistent with Hall–Petch-type strengthening trends [161,163,164,165,166]. However, this lamellar morphology is typically harmful to ductility, because stress concentrates at κ/α interfaces to facilitate microvoid nucleation and crack propagation, particularly when the carbide fraction is high, which can increase with Al-enhanced eutectoid carbon content [137,167,168]. By contrast, nanoscale intragranular κ′ precipitates can significantly increase the yield strength while preserving a comparatively good ductility, provided that the precipitates remain fine, coherent and well dispersed; strengthening arises from a combination of precipitation hardening, coherency strain hardening, and order strengthening that impedes dislocation motion [143].

At the dislocation scale, κ′ strengthening depends on whether dislocations shear the ordered precipitates or bypass them by Orowan looping. TEM evidence shows precipitate shearing can occur under planar slip conditions [123], whereas other studies identify Orowan bypass as dominant for rod-shaped κ′ or when precipitates are sufficiently large [140,141,169,170,171]. Yao et al. [123] described the operative mode as a competition governed primarily by precipitate size (radius *r*) and the antiphase boundary energy (γ_APB_) of the ordered precursor (L1_2_). In essence, very small κ′ particles are more likely to be sheared, but beyond a critical size the stress required for shearing becomes larger than that required for dislocation bowing, and the mechanism transitions to Orowan bypassing; once bypassing dominates, further coarsening generally reduces strengthening because inter-particle spacing increases. Kim et al. [172] experimentally supported such a transition and reported a critical radius on the order of ~13.4 nm for the switch from shearing to bypassing in duplex low-density steels. Coherency strengthening can additionally be expressed in terms of lattice misfit strain at the coherent κ′/γ interface; a representative form used in the literature is [143]

(4) $${\Delta \sigma }_{cs}=KM{\left(G\varepsilon_{c}\right)}^{\frac{3}{2}}\sqrt{\frac{rf}{0.5Gb}}$$

where *K* is a constant, *M* the Taylor factor, *G* the shear modulus, *b* the Burgers vector, *ε*_c_ the coherency misfit strain, *r* particle size, and *f* is precipitate volume fraction. As κ′ coarsens, misfit accumulation and interfacial energy can drive partial loss of coherency, further accelerating the transition toward bypassing and reducing the desirable coherent strengthening contribution.

In summary, κ-carbide is a double-edged constituent in Fe–Mn–Al–C lightweight steels: nanoscale coherent intragranular κ′ is generally the preferred strengthening state, whereas coarse and/or intergranular κ* is typically associated with ductility loss and premature fracture. The central processing challenge is therefore not “whether κ forms” but how to control its pathway, location, and scale through carefully designed composition–processing coupling.

#### 4.1.2. B2-Phase Precipitation and Strengthening

In Fe–Mn–Al–C low-density steels, relatively high Al additions (typically within ~10–32 wt% across lightweight-steel design space) are central to density reduction, but they also promote the formation of an ordered FeAl-type B2 superlattice that develops from the α-ferrite matrix [8]. Early studies therefore treated B2 primarily as a brittle intermetallic constituent and reported a pronounced ductility loss when B2 becomes excessive (e.g., when the volume fraction exceeds ~6.8%) [173]. At the same time, Al also increases the driving force for κ′-carbide precipitation; because κ′ is generally coherent and often shearable, dislocation cutting can reduce work hardening capacity and, in unfavorable cases, contribute to strain localization and boundary-related embrittlement. These concerns motivated the traditional “B2 suppression” paradigm. More recently, however, the design philosophy has shifted toward treating B2 as a controllable strengthening phase, provided that its size, morphology, and spatial distribution are carefully engineered [81].

**Figure 10:**
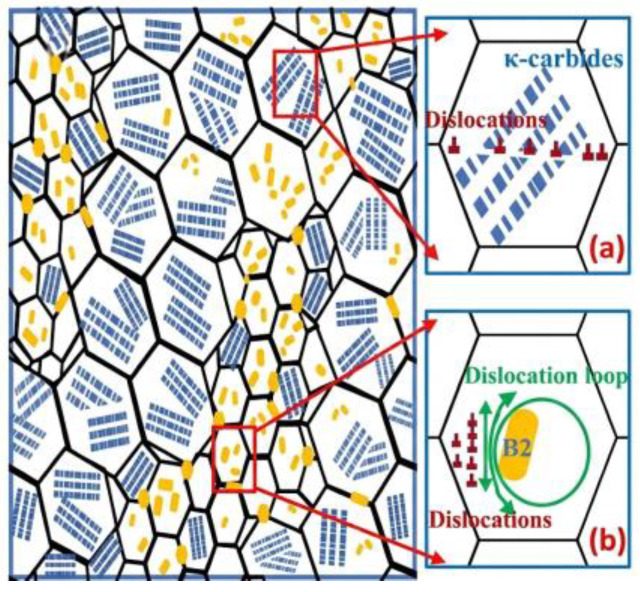
Schematic diagrams of precipitation strengthening mechanism: (**a**) dislocations shearing κ′-carbide; (**b**) dislocations bypassing B2 phase. (Reprinted with permission from [138], copyright MDPI, 2024).

The key distinction between κ′- and B2-based strengthening lies in the dislocation–precipitate interaction mechanism. Whereas κ′ is frequently shearable, the strengthening effect in so-called high-specific-strength steels (HSSS) relies strongly on the non-shearable character of B2 precipitates, which is associated with their largely incoherent interfaces with the matrix [93]. When glide dislocations encounter B2 particles, they are forced to bypass them via the Orowan mechanism, leaving dislocation loops around the particles [44,46]. The accumulation of such loops increases back stress and promotes dislocation pile-ups, which in turn encourages activation of additional slip systems and progressively refines slip band spacing during deformation. As a result, a microstructure strengthened by a fine and well-dispersed B2 population can exhibit markedly enhanced work hardening capacity compared with a κ′-dominated, shearable-precipitate regime (schematically illustrated in Figure 10) [138].

**Figure 11:**
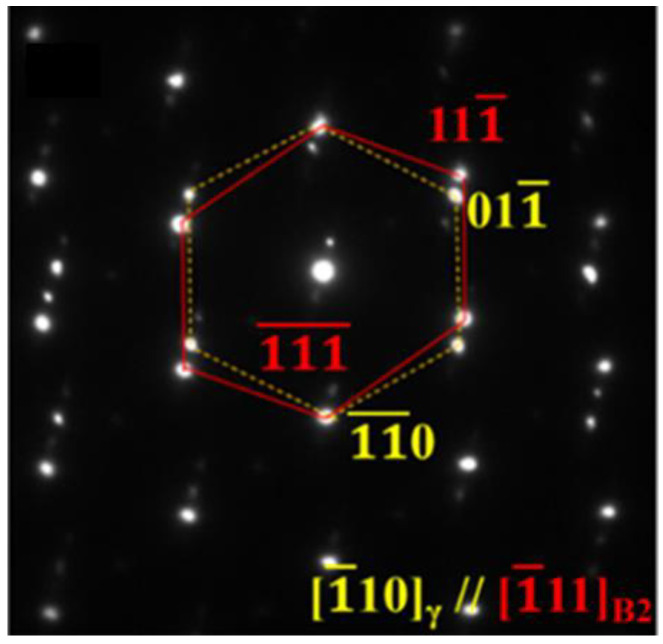
The orientation relationships with adjoining B2: SADP and diffraction spots of austenite and B2 are marked by dashed and solid lines, respectively. (Reprinted with permission from [138], copyright MDPI, 2024).

Because B2 can be either beneficial or highly detrimental depending on its morphology, microstructural control of B2, particularly particle size, aspect ratio, and dispersion uniformity, is central to balancing strength and ductility [46,86,93,101]. In many reported routes, B2 precipitation is achieved through hot/cold rolling followed by annealing at temperatures above ~900 °C, where deformation substructures provide potent heterogeneous nucleation sites during annealing [20,44]. In annealed HSSS, B2 has been reported to appear in several characteristic morphologies, including (i) coarse stringer bands aligned with the rolling direction (width 1–20 μm), (ii) intergranular polygonal particles (200–700 nm), and (iii) intragranular precipitates within austenite grains (50–500 nm) [44,47,93,174,175,176]. To reduce interfacial energy, these B2 precipitates often adopt specific orientation relationships with the surrounding austenite matrix, most commonly Kurdjumov–Sachs (K–S) or Nishiyama–Wassermann (N–W), as evidenced by TEM observations (Figure 11) [93].

A challenge is that the most detrimental B2 morphology, namely, coarse B2 stringers, is often inherited from upstream solidification and early thermomechanical processing. In high-Al steels, δ-ferrite can form readily during solidification and may persist due to incomplete peritectic transformation; upon cooling, δ-ferrite can order into B2, and subsequent rolling elongates these primary constituents into stringer structures along the rolling direction [95]. Subsequent annealing (typically near ~900 °C) may further generate secondary B2 precipitates, whose dispersion quality depends strongly on the prior deformation state and recrystallization progress, because stored-energy heterogeneities provide preferred nucleation sites for B2 [44,93,95,175,177,178].

From an engineering perspective, coarse B2 stringers are closely associated with brittle fracture tendencies and a strong anisotropy in tensile ductility, and therefore constitute a primary microstructural feature to be eliminated or minimized [86,93]. Accordingly, current optimization strategies aim to suppress banded B2 while promoting a high number density of fine and uniformly distributed B2 precipitates, typically through coordinated composition–processing design. While high Al helps ensure sufficient B2-forming tendency, elevated Mn and C are often discussed as beneficial for suppressing excessive δ-ferrite formation and, consequently, reducing the inheritance pathway that leads to coarse B2 bands [46,86]. Processing control, especially the degree of deformation, annealing temperature, and short-time annealing to retain selected unrecrystallized substructures, can further bias the system toward fine B2 precipitation on deformation bands rather than coarsening into boundary-decorated or banded morphologies. In this context, Hwang et al. [93] highlighted the strengthening contribution of unrecrystallized regions in HSSS: by shortening annealing at 900 °C from 15 min to 2 min, a heterogeneous structure with ~30% unrecrystallized austenite was retained, where high dislocation density and fine plate-like B2 precipitates along deformation bands produced strong substructure strengthening and Orowan strengthening. This yielded a very high yield strength (~1588 MPa), substantially exceeding that associated with fully recrystallized regions (~983 MPa).

#### 4.1.3. Multiphase Precipitation

Multiphase precipitation strengthening in duplex low-density steels aims to introduce effective precipitates in both constituent phases. Achieving this combined precipitation response requires relatively strict composition control. On the one hand, κ-carbide precipitation in austenite generally needs sufficiently high Al and C levels (>7 wt% Al and >0.8 wt% C), while on the other hand the formation of the DO3 superlattice (ordering) inside the B2 phase is favored within a higher Al range (roughly 12–22 wt% Al) [8,25]. Under such compositional conditions, aging treatments can generate a hierarchical arrangement of nanoscale ordered particles, enabling simultaneous strengthening of both γ- and B2-containing regions.

**Figure 12:**
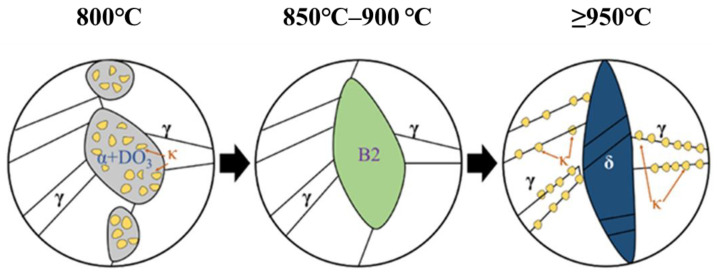
Schematic illustration of the secondary phases’ transformations at 800–950 °C. (Reprinted with permission from [157], copyright MDPI, 2024).

As shown in Figure 12, aging at 800 to 950 °C led to a slight coarsening of nanoparticles from 2 nm to 4 nm, while maintaining a fine dispersion [93,157]. This size range is still sufficiently small to provide strong resistance to dislocation motion, and the ordered nature of the precipitates contributes to additional strengthening through order-related effects. The reported mechanical response for this hierarchical microstructure reached a yield strength of ~1.6 GPa with ~20% ductility [93]. Compared with B2-strengthened HSSS at similar elongation, the yield strength was higher by ~240 MPa, and it also exceeded the peak yield strength of conventional κ′-strengthened austenitic lightweight steels by ~150 MPa [44,86,174,175,176]. Importantly, this strategy is discussed as a practical route to reduce reliance on coarse intergranular κ* films by shifting strengthening toward fine intragranular precipitation in both phases.

### 4.2. Refinement Strengthening

Grain refinement is one of the most effective approaches to improving the strength–ductility balance in duplex low-density steels because grain boundaries provide strong barriers to dislocation motion and also help distribute strain more uniformly between ferrite and austenite. In practice, grain refinement is achieved either by severe plastic deformation followed by controlled annealing, or by using transformation/recrystallization interactions to restrict grain growth. The key challenge is not only to make grains small, but also to maintain a stable and compatible duplex microstructure so that refinement does not trigger brittle boundary precipitation or early damage.

The importance of annealing control after deformation was clearly demonstrated by Sohn et al. [104]. In Fe–0.7C–12Mn–5.5Al steel, annealing at 670 °C for 10 min suppressed grain growth and produced very fine ferrite (~0.44 μm) and fine austenite (~1.92 μm). When the annealing temperature increased to 750 °C, both phases coarsened (ferrite ~0.88 μm; austenite ~3.00 μm), and the yield strength dropped sharply from 1100 to 740 MPa. This comparison highlights that, for duplex low-density steel, the processing window must be selected to balance recrystallization and grain growth.

Several studies have proposed refinement concepts that exploit phase transformation to constrain recrystallization. Zhang et al. [179] introduced a “transformation-constrained recrystallization” approach during annealing in the intercritical region. In this concept, the γ→α transformation generates submicron ferrite, and these ferrite regions pin migrating austenite boundaries during recrystallization, limiting the austenite grain size to 1.12 μm. Mohamadizadeh et al. [180] showed that under hot deformation at high strain rates, flow localization can form micro-shear bands that store high distortion energy. This locally increases the driving force for γ→α transformation and can enable ferrite nucleation even above the equilibrium transformation temperature; because growth is spatially confined within shear bands and deformation time is short, ferrite remains submicron (average ~0.3 μm). These results indicate that strain localization, usually considered undesirable, can be used in a controlled way to refine microstructure when transformation and growth are properly constrained.

Non-conventional processing routes have also been explored to obtain ultrafine duplex microstructures. Kumar et al. [120] reported that electropulsing treatment (EPT) on cold-rolled Fe–18Mn–10.5Al–1C–6Ni produced much finer microstructures than conventional furnace annealing. The proposed reasons include very high effective heating rates and current-assisted effects that accelerate diffusion and nucleation, so that recrystallization proceeds rapidly while grain growth is limited by the short treatment time and rapid cooling. In addition, EPT was reported to accelerate spheroidization of coarse B2 morphologies, converting lamellar B2 features into finer, more dispersed particles, which is beneficial for ductility while retaining strengthening contributions from hard phases. Although such current-assisted processing still needs further validation for scalability and process stability, it provides an alternative pathway for microstructure refinement in highly alloyed systems.

Besides grain size, substructure refinement also provides an important strengthening contribution in duplex low-density steels. Sohn et al. [108] noted that Taylor lattice structures in austenite can act as an internal subdivision mechanism. Carbon partitioning into austenite increases friction stress and tends to suppress cross-slip, promoting planar slip and the formation of dense dislocation walls rather than conventional dislocation cells. These dislocation walls subdivide austenite grains and reduce the dislocation mean free path, contributing to high strength in a way analogous to grain boundary strengthening. Similarly, deformation-induced low-angle boundaries (LAGBs) generated during cold rolling can strongly increase flow stress. Abedi et al. [181] showed in Fe–18Mn–8Al–0.8C duplex steel that increasing cold rolling reduction from 10% to 60% led to strong refinement mainly through dislocation rearrangement and substructure evolution rather than recrystallization. In ferrite (higher SFE), cross-slip-assisted recovery promotes cell formation and progressive sharpening into LAGBs, fragmenting grains into subgrains of ~500 nm. In austenite (lower SFE), planar slip produces dense dislocation microbands, while twin boundaries progressively lose their ideal coincidence character due to dislocation trapping and become effective barriers to slip. These deformation-induced boundaries and substructures provide an additional and often substantial strengthening increment, especially when subsequent annealing is designed to retain part of the substructure while restoring sufficient ductility.

## 5. Deformation Behavior

### 5.1. The TRIP Effect

Transformation-induced plasticity (TRIP) and TWIP are widely recognized as effective mechanisms to improve the strength–ductility balance in steels. In many austenite-containing alloys, the dominant deformation mode is often discussed in terms of the SFE of austenite: TRIP is frequently associated with low SFE (typically < ~20 mJ·m^−2^), whereas TWIP is more likely when SFE lies in an intermediate range (roughly ~20–40 mJ·m^−2^) [5,182,183]. For duplex low-density steels, however, SFE alone is not always sufficient to predict deformation behavior, because phase fraction, austenite stability, grain size distribution, and local constraint from the ferrite matrix can strongly modify the transformation tendency.

In duplex low-density steels, the TRIP effect primarily arises from DIMT, which relaxes local stress concentrations and provides additional strain hardening. The transformation is commonly described as a sequential path γ(fcc)→ε(hcp)→α′(bcc/bct) [182]. DIMT occurs when external deformation supplies enough driving force for metastable retained austenite to transform. The mechanical benefit comes from two coupled contributions: newly formed martensite increases the instantaneous flow stress because it acts as a hard second phase, while the transformation strain (volume expansion and shear) helps accommodate local incompatibility and delays strain localization in adjacent softer regions (often ferrite) [183,184]. As a result, TRIP-assisted steels often show a characteristic increase or “bulge” in work hardening rate, reflecting the competition between transformation-driven strengthening and local stress relaxation during deformation.

The effectiveness of TRIP depends strongly on the mechanical stability of retained austenite, which is sensitive to composition, grain size, and morphology [184,185,186]. Cai et al. [183] used an Olsen-type description to quantify austenite stability during straining:

(5) $$f_{\gamma }=f_{\gamma 0}e^{-k\varepsilon }$$

where *f*_γ0_ is the initial austenite fraction, *f*_γ_ is the austenite fraction at strain *ε*, and *k* characterizes mechanical stability (a larger *k* indicates lower stability and faster transformation) [183,187]. Experimentally, higher annealing temperatures are often associated with reduced austenite stability, which is typically attributed to the combined effects of austenite grain coarsening and solute partitioning [183,188,189]. Grain refinement can stabilize austenite mechanically; for example, Takaki et al. [190] elucidated that reducing austenite grain size can change the transformation mode and raise the elastic strain energy barrier for martensitic transformation, thereby suppressing DIMT. In contrast, grain growth at higher annealing temperatures reduces this barrier and facilitates transformation.

Composition effects on austenite stability in duplex low-density steels require attention to diffusion kinetics and partitioning paths. Mn and C both stabilize austenite, but their influence differs because C redistributes rapidly, while Mn diffusion is slower and vacancy-mediated [191,192]. Zhang et al. [189] emphasized that partitioning should be viewed in two stages: partitioning at high temperature that sets phase fractions, followed by redistribution during cooling or lower-temperature holding. Because higher annealing temperatures often increase the austenite fraction, carbon can become diluted in austenite, reducing its stability and promoting TRIP during deformation [183,189]. In many cases, the stability trend with annealing temperature/time is therefore strongly governed by the carbon content in austenite, rather than by Mn partitioning alone.

Retained austenite (RA) stability is also affected by morphology and crystallographic orientation. RA in duplex microstructures is commonly heterogeneous, including blocky austenite at triple junctions and lath-like austenite within grains or along phase boundaries [193]. While the RA fraction may vary non-monotonically with annealing temperature (reflecting the balance between reverse transformation and thermally induced martensite formation during cooling), the propensity for DIMT during loading can depend on the Schmid factor (SF) relative to the loading direction [183,186]. Grains with higher SF tend to deform more readily; during early deformation, lattice rotation often increases SF to facilitate slip, whereas a later decrease in average SF can coincide with the onset of TRIP because high-SF grains are preferentially consumed by martensitic transformation [188,194]. In this context, blocky RA, often subjected to higher triaxial stresses at boundaries and frequently associated with high SF, tends to transform earlier, while lath-like RA is more constrained by the surrounding matrix and may require more rotation or higher local strain before transforming [186,195].

**Figure 13:**
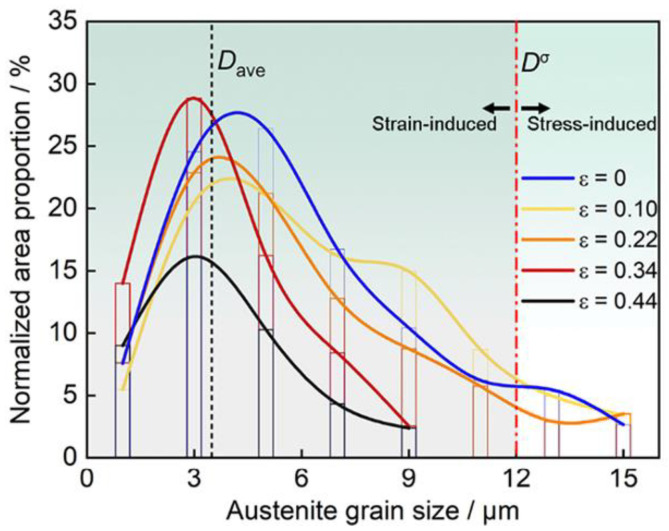
The distribution of austenite grain size in the specimens deformed at different strain amounts. *D*_ave_ means the average grain size of austenite before deformation, and *D*_σ_ means the critical grain size of austenite for stress-induced martensite transformation at ambient temperature. The amount of austenite before deformation was set as 1, and it decreases according to the variation in austenite fraction. (Reprinted with permission from [188], copyright Elsevier, 2022).

Recent studies have further shown that a strict SFE-based criterion does not always capture TRIP activation in duplex steels [184,188,196,197]. For example, Chen et al. [188] reported TRIP-dominated behavior even in a duplex steel with a relatively high SFE (~53 mJ·m^−2^), where conventional SFE-based expectations would suggest TWIP or dislocation glide as dominant. To better account for such cases, grain-size-based stability concepts have been proposed, such as a critical austenite size *D*_σ_ framework that distinguishes transformation in coarse grains from transformation in fine grains [198,199,200]. In general terms, coarse austenite (*D* > *D*_σ_) may transform under stress in the elastic regime and contribute less to the ductility improvement, whereas finer austenite (*D* < *D*_σ_) tends to transform during plastic deformation, which is more beneficial for the TRIP effect and uniform deformation [188,201,202,203]. Consistent with this interpretation, the austenite grain-size distribution evolves with strain as transformation preferentially consumes a subset of grains (Figure 13).

### 5.2. The TWIP Effect

**Figure 14:**
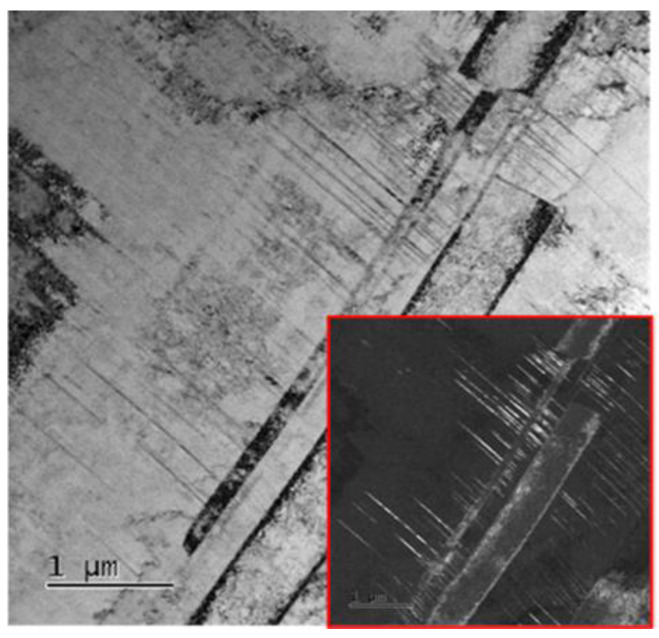
A bright-field TEM image exhibiting fine deformation twins; the inset at the bottom right is the corresponding DF image. (Reprinted with permission from [209], copyright MDPI, 2025).

The TWIP effect is associated with the formation and growth of nanoscale deformation twins in the austenite matrix, as illustrated in Figure 14. Unlike TRIP, which involves a phase transformation, TWIP is better described as a substructure evolution process. Aristeidakis et al. [204] proposed a physically based view in which twins develop as the density and arrangement of planar defects evolve during deformation. At the crystallographic level, twin embryos are commonly related to the dissociation of perfect dislocations into Shockley partials on {111} planes, forming stacking fault packets on successive close-packed planes that can develop into twins [205,206,207]. Twinning kinetics include nucleation, lengthening, and thickening, which is strongly influenced by the SFE. Lower SFE increases partial dislocation separation and reduces the barrier for twin nucleation, thereby promoting TWIP. As twins multiply, twin boundaries act as effective obstacles to dislocation motion and progressively subdivide the austenite grains, reducing dislocation mean free path and generating back stress. This effect is often referred to as a “dynamic Hall–Petch” contribution and is a key reason why TWIP steels can sustain high strain hardening rates at large strains [208].

**Figure 15:**
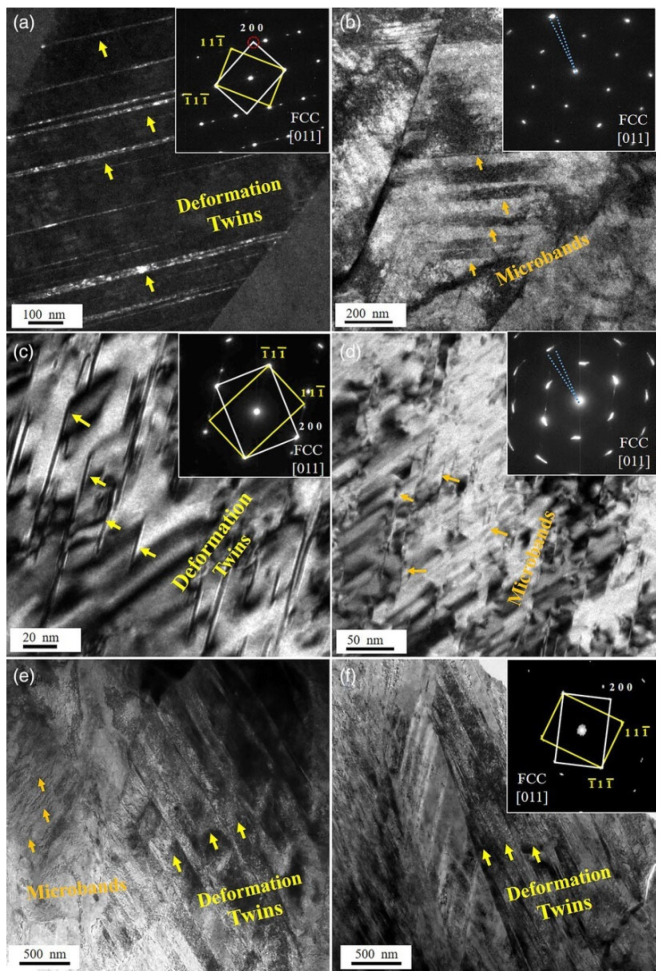
The bright-field TEM images and the corresponding SAED patterns of a Fe–13.6Mn–1.2Al–0.6C steel at different strains of (**a**,**b**) 30%; (**c**,**d**) 50%; and (**e**,**f**) fracture. The zone axis in (**a**–**f**) are along the [110] fcc direction. (Reprinted with permission from [215], copyright Wiley, 2023).

Although both TRIP and TWIP are affected by austenite grain size and orientation, they often show a sequential or hierarchical activation. In duplex low-density steels, experimental observations frequently indicate that TRIP contributes more at early deformation stages, while twinning becomes more important at higher strains after a portion of the unstable austenite has already transformed [188]. From an SFE perspective, twinning is generally considered favorable over a relatively broad range (~18–80 mJ·m^−2^ in related alloy systems), whereas lower SFE tends to favor *ε*-martensite formation and higher SFE promotes planar or wavy dislocation glide rather than twinning (Figure 15) [8,210,211,212,213,214,215]. In practical terms, the best work hardening response in duplex steels often comes from the coordinated operation of dislocation slip, TRIP (where applicable), and then TWIP at higher strains, rather than from a single dominant mechanism across the full strain range.

To move beyond descriptions based on a single average twin spacing, Gutierrez-Urrutia et al. [182] classified deformed austenite grains into three categories according to crystallographic orientation and corresponding twinning behavior, providing a more quantitative link between texture, substructure evolution, and work hardening. The rationale is that orientation affects resolved shear stress (Schmid law) and the way dislocations interact with twins [216]. Grains near <001>//tensile axis tend to twin less and deform mainly by dislocation glide, often showing dislocation cell refinement (Type I). Grains near <111>//tensile axis more readily activate multiple twinning systems, forming a dense and highly subdivided twin network (Type III). The intermediate group (Type II) often shows a lamellar twin structure: a primary twin system may be activated, but the resulting substructure and latent hardening can suppress further twinning on secondary systems [182]. This framework is useful because it allows for composite mean-free-path models that treat different obstacle types in different grain families (e.g., cell size in Type I, twin spacing in Types II/III), improving the physical basis for work hardening descriptions in TWIP-active microstructures.

TWIP/TRIP competition is also influenced by local orientation and microstructural heterogeneity within a single austenite grain. For instance, Sohn [106] observed the case where deformation twins and α′-martensite formed concurrently within austenite in Fe–8.5Mn–5.6Al–0.8C after annealing at 900 °C for 30 min, and related this to orientation-dependent deformation. Martensite transformation tends to initiate in domains with a higher effective driving force for slip/strain localization (often associated with higher Schmid factor regions such as near boundaries where dislocation pile-ups can raise local stress), whereas twins can preferentially form in regions with comparatively lower Schmid factor under the same macroscopic loading state [106,214,217]. These observations support the view that TWIP and TRIP can coexist locally, but their spatial activation is controlled by heterogeneous stress/strain partitioning and orientation.

A strong grain size dependence is also widely reported. Coarser austenite grains tend to show TRIP more readily, while finer grains—often cited below ~10 μm—more commonly show TWIP-dominated deformation [218]. In early deformation, coarse austenite grains with a lower stability can transform first. As transformation subdivides coarse grains and consumes the most unstable population, the remaining austenite increasingly accommodates strain by twinning rather than further martensitic transformation [188]. Even though grain refinement can raise the critical stress for both twinning and transformation, the competition between them does not scale identically. In fine grains, the elastic strain energy penalty for martensitic transformation becomes more significant, and the stress required to trigger TRIP can exceed the stress needed for twinning; therefore, at higher strains, twinning can become the preferred accommodation mode in the refined austenite [219,220,221]. From a design standpoint, this implies that controlling the austenite grain size distribution is a practical lever to tune whether the alloy exhibits early TRIP, late TWIP, or a mixed response.

Most TWIP discussions focus on austenite, but twinning in ferrite has also been reported in certain Fe–Mn–Al-based duplex steels and deserves consideration when interpreting deformation partitioning. Nezhadfar et al. [222,223] investigated TWIP-like behavior in ferrite for Fe–0.07C–11.15Mn–5.6Al–0.12Si and related ferrite twinning to the dissociation behavior of screw dislocations in the bcc lattice under specific alloying and stress/temperature conditions [222,223,224,225,226,227]. In their description, dislocation dissociation and the motion of partials on adjacent {112} planes can alter local stacking, leading to multilayer stacking faults and eventually observable twins. Interestingly, they reported that twin frequency increased with temperature in a warm deformation regime and suggested that thermal activation assisted partial dislocation motion and dissociation, promoting twinning in this specific alloy system [222]. Twinning in ferrite at ambient temperature has also been reported. For example, Saberipour et al. [87] argued that ferrite twinning cannot be attributed to grain size and Schmid factor alone; instead, the combined effects of ferrite grain size, solute/solute–interstitial interactions, and a double-cross-slip mechanism can enable twin nucleation and thickening under certain conditions [87,224,228]. In this picture, high Al and interstitials assist dissociation of a/2<111> dislocations into partials, while solute pinning promotes bowing and cross-slip onto adjacent {112} planes, providing a pathway for twin development.

### 5.3. Dislocation Slipping

Dislocation glide in duplex low-density steels can show either wavy slip or planar slip, and the associated substructures differ markedly. In materials dominated by wavy slip, deformation at low to medium strains commonly produces dislocation cells and cell walls, while at higher strains, non-crystallographic microbands may develop. In contrast, planar-slip-dominated deformation tends to suppress dynamic annihilation through cross-slip, so dislocations accumulate on a limited number of slip planes and form planar dislocation arrays, dense dislocation walls, Taylor lattices, and microband-related structures. This difference is important because it strongly affects the work hardening and strain localization behavior.

The SFE of austenite is an important, but not exclusive, factor in determining the glide mode. Wavy slip is generally more likely at high SFE (>~40 mJ·m^−2^) because partial dislocations are closely spaced and cross-slip becomes easier, promoting three-dimensional dislocation motion [229]. However, many duplex low-density steels show pronounced planar slip even when the nominal SFE is relatively high. This behavior is often explained by “slip plane softening”, where localized obstacles on a slip plane make it energetically favorable for subsequent dislocations to follow the same plane rather than to cross-slip. Such obstacles can originate from short-range ordering (SRO) or shearable coherent precipitates (often associated with κ-related ordering/precursors), which impose an energy barrier on the leading dislocation; dislocations then pile up and assist the leading dislocation to cut through, making later glide on the same plane easier and thus promoting planar slip [175,230,231]. Planar slip has also been reported in high-SFE duplex steels without clearly resolved κ precipitates, and has been attributed to Mn–C SRO, dislocation pile-ups, or strong solute–dislocation interactions that restrict cross-slip [232,233,234]. In particular, Zhang et al. [90] emphasized that high concentrations of substitutional solutes (Mn, Al) together with interstitial C can generate large friction stress and solute drag, effectively pinning dislocations and increasing the stress required for screw dislocations to cross-slip. This solute-drag picture is consistent with Kocks-type concepts in which interstitials act as continuous locking points along dislocation lines, thereby confining motion to primary planes and favoring planar slip even at high SFE [235].

When planar slip dominates, several characteristic substructures may appear, often in sequence as strain increases. A representative structure is the Taylor lattice, which consists of organized planar slip bands from different systems that intersect and form a regular dislocation wall network [22]. The Taylor lattice typically develops when a primary planar slip system is activated first (commonly on {111} Effect of Mn and in austenite), followed by the activation of additional systems; intersections between primary and secondary planar bands generate a stable lattice-like arrangement of dislocation walls that progressively subdivides the grain. Song et al. [22] compared hot-rolled and annealed conditions and reported that the initial stored energy and the operative slip mode strongly influence whether the material evolves toward dislocation cell structures or Taylor lattices. In the hot-rolled state, high stored energy facilitates cross-slip and rapid formation of entangled dislocation cells and dense cell walls, which can raise yield strength but tends to cause earlier work hardening saturation and limited ductility [236,237]. After stress relief annealing, planar slip on multiple systems becomes more active, enabling Taylor lattice development; unlike pre-existing cells that mainly provide “static” strengthening, the progressive formation and refinement of Taylor lattices during straining acts as a dynamic barrier to dislocation motion and helps sustain work hardening to delay necking.

**Figure 16:**
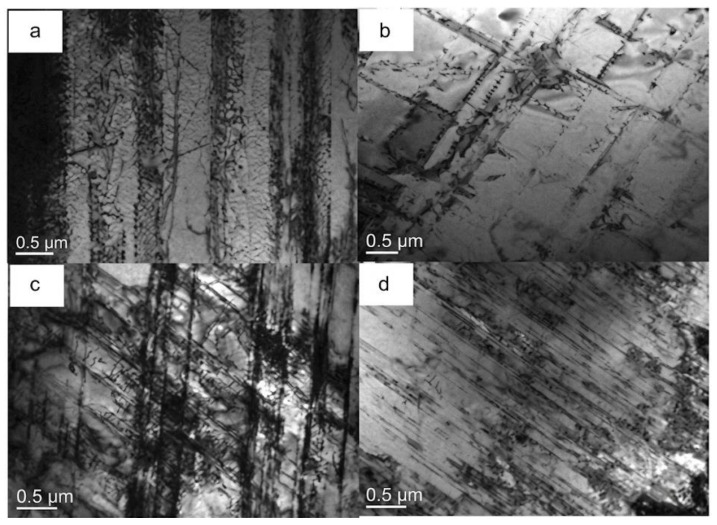
TEM image of microstructural evolution in an investigation Fe-26Mn-10Al-1.0C steel: (**a**) planar slip (ε = 0.05), (**b**) Taylor lattice (ε = 0.1), (**c**) crossing slip bands (ε = 0.3), (**d**) microbands (ε = 0.3). (Reprinted with permission from [33], copyright Wiley, 2013).

At larger strains, planar slip alloys often develop microbands, which represent a further stage of strain accommodation and grain subdivision. A commonly described sequence is that a Taylor lattice domain structure forms first; then, domain boundaries emerge between differently oriented Taylor lattice regions, typically as walls of geometrically necessary dislocations (GNDs) that accommodate lattice misorientation. With increasing strain, paired dislocation walls can form, and the region between them is identified as a microband. These microbands often align with active slip planes and can terminate at grain boundaries or domain boundaries; continued deformation increases microband density and interactions, promoting subgrain formation and effective grain subdivision [238,239,240]. Ding et al. [241] analyzed Fe–18Mn–10Al–*x*C and pointed out that microbands generally require sufficient accumulated plastic strain to develop; alloys that fracture early may show planar slip but limited microband formation. For Fe–18Mn–10Al–1.2C, they reported a three-stage work hardening behavior: planar slip dominates early; Taylor lattice and microbands develop at intermediate strain; and at higher strain, increased microband density and interactions help maintain work hardening (Figure 16) [241].

In addition to microband-driven subdivision, dynamic slip band refinement (DSBR) has been proposed as another planar-slip-controlled hardening mechanism. Welsch et al. [232] used electron channeling contrast imaging (ECCI)/TEM observations to show cases where microbands between paired dislocation walls were not evident, while dense slip bands progressively filled the grain. In the DSBR concept, dislocations pile up on a given slip plane and generate back stress on the operating source. As back stress increases, the source becomes less effective and may stop emitting loops when the local stress drops below the activation level; further deformation then requires activation of additional sources on nearby or intersecting slip systems, which effectively reduces the spacing between active slip bands and refines the slip pattern [232,242,243,244,245]. The associated strain hardening is commonly linked to the long-range “passing stress”, which scales with the inverse of the mean slip band spacing. A frequently used form is [246,247]

(6) $$\sigma_{SH}\left(\varepsilon \right)=K\cdot M\cdot G\cdot \frac{b}{D}$$

where *K* is a geometric factor, *M* the Taylor factor, *G* the shear modulus, *b* the Burgers vector, and *D* is the mean spacing between slip bands. Within this framework, *D* becomes a practical microstructural parameter for describing work hardening in planar-slip-dominated steels [232]. Grain size can also influence DSBR: refinement tends to trigger earlier source exhaustion and earlier activation of new sources, which can increase the initial hardening rate but may also exhaust the refinement potential sooner if the slip band spacing reaches a limiting value at a relatively low strain [141].

### 5.4. Coordination Deformation Between Austenite and Ferrite

The mechanical performance of duplex low-density steels is controlled not only by the intrinsic deformation mechanism of each phase (e.g., TRIP, TWIP, dislocation slip), but also by how austenite and ferrite share strain and transfer load. Because these two phases differ in crystal structure, slip behavior and hardening response, maintaining deformation compatibility across α/γ interfaces is critical for delaying localization and suppressing interface damage. This coordination depends on austenite stability, phase fraction and morphology, interface density, and the relative hardness of these two phases, all of which are strongly affected by processing.

In low-SFE duplex steels where TRIP is active, coordinated deformation can be strongly influenced by the sequence of austenite transformation. Cai et al. [183] studied a duplex steel with SFE below ~40 mJ·m^−2^ and described a stress relaxation process associated with discontinuous multi-stage martensitic transformation. At early strain, the softer ferrite tends to yield first. Because Mn partitioning and other heterogeneities can produce a spread in austenite stability, transformation does not occur uniformly; less stable austenite grains transform first [248]. The accompanying transformation strain and volume expansion locally compress the surrounding ferrite, forcing it to accommodate additional plastic strain and promoting local stress redistribution. In this way, sequential austenite transformation repeatedly activates ferrite deformation near transforming regions, helping to delay necking and improving the strength–ductility balance through a combination of transformation hardening and local stress relaxation.

In higher-SFE duplex steels, these two phases often exhibit different glide characteristics: ferrite tends to deform by wavy slip with dislocation cells and cell blocks, while austenite more readily shows planar slip and planar substructures as discussed above. In such cases, compatibility is often achieved through interface-mediated load transfer. During early deformation, ferrite may yield first, and strain mismatch is accommodated by dislocation accumulation at α/γ interfaces (e.g., high-density dislocation walls), which assists stress transfer into austenite and stabilizes overall plastic flow [249,250]. As strain increases, ferrite plays an important role in accommodating heterogeneous deformation near interphase boundaries of the harder phase. Processing can improve this coordination; for example, solution treatment or other routes that break up continuous ferrite bands into a more discontinuous morphology can help slip bands traverse ferrite regions and connect neighboring austenite grains, reducing strain localization. Failure often initiates when interface compatibility deteriorates, leading to the void formation at α/γ interfaces due to accumulated mismatch and limited accommodation capacity in the ferrite substructure at high strain. In representative observations, austenite substructure may evolve from planar arrays to Taylor lattices/high-density walls and then to microbands, while ferrite evolves from interacting dislocations to cells and eventually cell blocks [90].

The assumption that “the softer phase always carries more strain” does not always hold in duplex low-density steels. In some banded or heterogeneous microstructures, austenite can accommodate more strain than ferrite if ferrite grains are relatively coarse or if the ferrite substructure saturates early, which shifts further strain into austenite bands. More broadly, the relative hardness of austenite and ferrite is not fixed and can be reversed by microstructure design and test conditions. In conventional solution-treated conditions, ferrite often appears softer because austenite is strengthened by C and Mn partitioning (higher friction stress), whereas ferrite may contain more mobile dislocations and shows easier cross-slip due to its bcc structure and a higher effective SFE [90,235]. In contrast, Magagnosc et al. [251] reported a case where fine-grained ferrite acted as the harder phase and was distributed around coarse austenite grain boundaries; here, Hall–Petch strengthening in fully recrystallized ferrite led to a hardness inversion and a “composite” deformation response. Their neutron diffraction results indicated load transfer from early-yielding austenite orientations to elastic ferrite and harder austenite orientations, followed by co-deformation once ferrite yielded, giving a combined response in which ferrite contributed strength and austenite provided ductility and work hardening [251,252]. Gao et al. [253] innovatively utilized spherical nanoindentation to estimate the crystallographic orientation dependence of each constituent phase in a Fe-12Mn-8Al-0.8C duplex steel. The micro-scale stress–strain partitioning exhibits a dynamic evolutionary feature, transitioning from an interphase mismatch-dominated regime to one governed by crystallographic orientation. In the early stage of plastic deformation, the δ-phase acts as the hard phase and bears higher Mises stress. Conversely, the softer γ-phase yields preferentially and accommodates the majority of the plastic strain, resulting in highly concentrated strain near the phase/grain boundaries. As deformation progresses, the γ-phase displayed a stronger strain-hardening capacity, which leads to a rapid increase in its slip resistance increment. At this point, the micromechanical anisotropy induced by crystallographic orientation supersedes the phase volume fraction as the core factor governing stress–strain partitioning. Temperature can further shift this balance: ferrite often shows stronger thermal softening than austenite in bcc systems, consistent with Zerilli–Armstrong-type behavior, which can reduce the effectiveness of ferrite-based composite strengthening at elevated temperatures [254,255]. Similarly, if ferrite is strengthened strongly by hard particles (e.g., B2 nanoparticles), it may lose deformation capacity relative to austenite, increasing interface stress concentration and promoting final fracture once the compatibility is no longer maintained [250]. The deformation mechanisms mentioned above are summarized in Table 2.

## 6. Application Performance

From a perspective of materials engineering, service environments in sectors such as automotive, aerospace, and shipbuilding are often highly demanding. Typical challenges include intense salt spray exposure and chloride ion attack in marine atmospheres, corrosion of vehicles operating in winter de-icing salt conditions, and fatigue damage in structural components subjected to cyclic loading. In addition, atomic hydrogen generated during corrosion reactions, or hydrogen already present in the surrounding environment, can diffuse into steels and trigger hydrogen embrittlement. This can cause a sharp loss of toughness and ductility, and even lead to catastrophic, sudden fracture without warning. Therefore, developing an in-depth understanding of the three core performance dimensions of duplex-phase low-density steels, including corrosion resistance, fatigue behavior, and susceptibility to hydrogen embrittlement, is essential for their safe and reliable use in engineering applications [88].

### 6.1. Corrosion Resistance

The corrosion resistance of duplex-phase low-density steels largely depends on the passive film that forms on their surface. Because these steels contain a high level of Al, they can quickly develop a thin and dense Al_2_O_3_-rich passive layer in oxidizing environments. This alumina film is thermodynamically stable and chemically inert, so it effectively separates the corrosive medium from the underlying metal, giving the steel a good resistance to atmospheric corrosion. Studies show that the passive film is often a multilayer composite structure containing elements such as Fe, Al, and Mn. Its composition, thickness, and compactness are strongly affected by both alloy design (for example, adding Cr and Mo can improve film quality) and environmental factors (such as pH value, temperature, and ion species) [256].

Despite its generally good corrosion resistance, duplex-phase low-density steel is still prone to localized corrosion in environments containing aggressive ions, especially chloride ions (Cl^−^), and this is a major cause of structural failure. Pitting corrosion is a highly localized form of attack that starts when the passive film is damaged in specific areas by ions like Cl^−^. In duplex-phase low-density steels, pit initiation is often linked to microstructural inhomogeneity. For example, the potential difference across ferrite/austenite phase boundaries can cause micro-galvanic effects, leading to preferential dissolution of one phase; the passive film near non-metallic inclusions such as sulfides, or around κ-carbides, may be weaker and become preferred sites for Cl^−^ attack; and local segregation of elements such as Mn may reduce passive film stability and trigger pitting. Once a pit forms, hydrolysis of metal cations inside the pit creates an occluded environment that is acidic and enriched in Cl^−^, which accelerates pit growth in a self-sustaining manner and poses a serious threat to structural integrity. Adding Cr and Mo is an effective approach to improving pitting resistance.

Stress corrosion cracking (SCC) refers to cracking that occurs when a material is exposed to a specific corrosive medium while under tensile stress. For duplex-phase low-density steels, SCC is an important failure mode in marine atmospheres or Cl^-^, containing aqueous solutions. The mechanism is commonly understood as a combined effect of anodic dissolution (AD) and hydrogen-induced cracking (HIC) [257]. At the crack tip, tensile stress continuously exposes fresh metal surfaces, accelerating anodic dissolution and promoting crack growth. Meanwhile, electrochemical reactions at the crack tip generate hydrogen atoms. Driven by the stress gradient, hydrogen tends to accumulate in the high-stress region near the crack tip, reducing cohesive strength and promoting localized plastic deformation, thereby assisting crack propagation. It has also been reported that ordered phases such as the B2 phase may affect dislocation slip behavior and thus influence SCC susceptibility.

### 6.2. Hydrogen Embrittlement

Hydrogen embrittlement is a widespread and highly dangerous problem in high-strength steels. Because duplex-phase low-density steels combine high strength with a complex phase structure, their susceptibility to hydrogen embrittlement has attracted significant attention. The α/γ duplex-phase microstructure provides hydrogen with complicated paths for diffusion and redistribution. Ferrite (bcc) has larger interstitial sites and a more open atomic packing, so hydrogen diffuses through ferrite several orders of magnitude faster than through austenite (fcc). In contrast, austenite has a lower lattice distortion energy and a much higher hydrogen solubility than ferrite. As a result, in a duplex-phase structure, ferrite enables hydrogen to be transported rapidly into the interior, while austenite can store a large amount of hydrogen and reduce the concentration of freely mobile hydrogen in the matrix. This makes the macroscopic hydrogen diffusion behavior in duplex-phase low-density steels highly complex, governed jointly by the phase fraction, phase connectivity, and interfacial characteristics.

Recent studies have revealed the strong potential of microalloying to improve hydrogen embrittlement resistance in duplex-phase low-density steels. One systematic work compared the effects of V and Cu additions on the hydrogen embrittlement behavior of medium-Mn dual-phase lightweight steels and found that adding V led to the formation of finely dispersed V-rich carbides [88]. Thermal desorption analysis (TDA) showed that these carbides acted as high-energy hydrogen trap sites, significantly increasing the total trapped hydrogen and effectively reducing the amount of diffusible hydrogen, thereby improving resistance to hydrogen embrittlement. With Cu addition, Cu-rich precipitates formed, and Cu also segregated at phase boundaries and grain boundaries. These Cu-rich precipitates and segregation layers likewise served as deep hydrogen traps and may have hindered hydrogen diffusion along interfacial pathways. Experimentally, the Cu-containing steel exhibited the best hydrogen embrittlement resistance among the steels studied.

## 7. Challenges

Although duplex low-density steels show strong potential in achieving a favorable balance among strength, ductility, and density, several key barriers remain before reliable engineering application can be realized. In general, the major issues include limited quantitative understanding of governing mechanisms, insufficient service property data, difficulties in stable industrial production and quality consistency, and unresolved challenges in forming and joining.

### 7.1. Fundamental Mechanisms and Quantitative Description

The mechanical response of duplex low-density steels is highly sensitive to multiphase microstructures and their interfaces, including α/γ interphase boundaries, precipitate/matrix interfaces, and grain boundaries. These interfaces control dislocation transfer, strain partitioning, crack initiation, and crack growth. However, quantitative knowledge of interfacial segregation, atomic structure, and interfacial bonding remains limited, which restricts the development of consistent physical descriptions for strengthening and damage. Further work should combine APT and high-resolution TEM (HRTEM) to characterize interfacial features and link these observations with first principles calculations and molecular dynamics simulations to establish testable relations among interface structure, dislocation behavior, and damage evolution.

In addition, the micro-mechanism of the TRIP effect in duplex low-density steels requires further clarification. In particular, how Al affects austenite mechanical stability through its coupled influence on stacking fault energy, solid solution strengthening, and carbon partitioning is not yet captured by a robust quantitative model. Future studies should build a coupled description connecting composition, austenite stability, transformation kinetics, and macroscopic mechanical response, and relate this framework to practical heat treatment paths and microstructural states to improve predictability in alloy and process design.

### 7.2. Service Properties and Environmental Sensitivity

Compared with tensile properties, engineering applications require systematic evaluation of service-related performance. Application-relevant properties of Fe-Mn-Al-C low-density steels remain insufficiently understood due to limited experimental datasets, motivating more comprehensive, mechanism-guided studies. Building on our previous work on austenitic low-density steels [258], we will further extend our efforts to duplex (α + γ) low-density steels. Although recent studies have reported microstructural conditioning and nine strain-rate-dependent tensile responses in duplex low-density steels, systematic data on fatigue and fracture toughness are still limited, and clear trends across different microstructural states, temperatures, and strain rates have not been established. These properties are essential for reliability assessment and structural design in automotive and aerospace components.

Hydrogen embrittlement and corrosion resistance also deserve priority. The high density of interfaces and dislocations in duplex low-density steels may provide both trapping sites and transport paths for hydrogen, increasing susceptibility in hydrogen-containing environments. Therefore, systematic studies on property degradation under different hydrogen sources and loading conditions are required, together with alloy and microstructure strategies to reduce hydrogen embrittlement sensitivity. In parallel, the high Al and Mn contents significantly modify electrochemical behavior. While Al may promote passive film formation, micro-galvanic effects may accelerate localized corrosion. Corrosion behavior and SCC susceptibility should be evaluated in relevant environments (e.g., salt spray and acidic media) to define practical application limits.

### 7.3. Industrial Manufacture

Industrial production of duplex low-density steels is first constrained by process control during melting and casting. Reactive elements such as Al and Mn readily react with oxygen and nitrogen in the melt, forming inclusions (e.g., Al_2_O_3_ and MnO) that reduce cleanliness and increase cracking risk during subsequent processing. Protective melting practice, vacuum refining, and clean casting routes tailored for this alloy system are therefore essential to achieve stable quality.

Moreover, due to the high alloy content, macro- and micro-segregation of Mn, Al, and C during solidification is more pronounced, leading to non-uniform microstructure and properties across the cast product. Mitigating segregation through continuous casting control combined with an appropriate homogenization heat treatment is important for batch-to-batch consistency. In addition, high Mn contents reduce thermal conductivity, increasing temperature gradients during heating/cooling and raising thermal stress and hot-cracking risk. This places higher demands on reheating schedules, cooling strategies, and process control accuracy.

### 7.4. Hot Working, Cold Forming and Welding

Because phase transformation and precipitation are complex in duplex low-density steels, the hot working window can be relatively narrow, and edge cracking or surface cracking may occur during hot rolling or hot forming. Hot deformation simulation and process modeling are needed to define workable processing windows, and to clarify how deformation temperature, strain rate, and cooling path affect microstructural evolution and hot ductility.

For cold forming, duplex low-density steels can show high elongation, but high strength and high work hardening rates increase forming loads and springback, making dimensional control difficult. Accurate forming models capturing constitutive behavior together with phase stability and hardening features are required, and process/tooling optimization is needed to reduce springback and fracture risk.

Welding remains another key barrier. Welding thermal cycles may disrupt the optimized duplex microstructure and precipitation state, leading to brittle constituents in the heat-affected zone (e.g., coarse κ carbides or intermetallic phases). In addition, Al oxidation/evaporation and hot cracking sensitivity associated with high Mn further complicate welding. Therefore, welding consumables and joining processes compatible with duplex low-density steels microstructural stability (e.g., laser welding and friction stir welding) should be developed, together with systematic evaluation of joint strength, toughness, and fatigue performance.

## Figures and Tables

**Table 1:** Microstructures and mechanical properties at different annealing temperatures [47].

Alloy		500 °C	1050 °C
Ni-containing steel	Matrix phase	γ	γ + α
	Precipitations	Cuboidal κ-carbide(<100 nm) + Coarse B2 particles (2–7 nm) + B2 stringer bands	Coarse B2 particles (2–4 nm) + Disk-like B2 phase (~200 nm)
	Properties	UTS: 960 MPa Elongation: 10.2%	UTS: 1350 MPa Elongation: 22%
Ni-free steel	Matrix phase	γ + α	γ + α
	Precipitations	Cuboidal κ-carbide(<90 nm) + needle-like κ-carbide + Coarse B2 particles (2–4 nm)	Coarse B2 particles (2–4 nm) + Disk-like B2 phase (~200 nm)
	Properties	UTS: 840 MPa Elongation: 11.3%	UTS: 1040 MPa Elongation: 25%

**Table 2:** The summary of deformation mechanism and corresponding impacts on duplex lightweight.

Deformation Mechanism	Core Characteristics	Impacts on Duplex Steel
TRIP Effect	Metastable austenite undergoes DIMT during deformation and newly formed martensite acts as a hard secondary phase.	Improves strain hardening rate and realizes strength-ductility synergy; relieves local stress concentration and reduces microcrack initiation risk.
TWIP Effect	Nanoscale deformation twins form in the austenite matrix; twin boundaries act as dislocation barriers and realize dynamic grain refinement.	Twin-induced grain subdivision enhances strain hardening capacity without sacrificing plasticity.
Dislocation Slipping	Planar slip: Dislocations move along limited slip planes, forming Taylor lattices and microbands. Wavy slip: Dislocations move three-dimensionally, forming dislocation cells and cell walls.	Planar slip: Increases yield strength and strain hardening rate with moderate ductility. Wavy slip: Achieves good ductility with lower strength improvement.
Coordinated Deformation	Stress redistribution occurs at α/γ interfaces; maintains deformation compatibility between α and γ.	Enhances formability and reduces local cracking during plastic deformation; realizes uniform stress distribution and delays strain localization.

## Data Availability

No new data were created or analyzed in this study. Data sharing is not applicable to this article.
